# Linking Aβ42-Induced Hyperexcitability to Neurodegeneration, Learning and Motor Deficits, and a Shorter Lifespan in an Alzheimer’s Model

**DOI:** 10.1371/journal.pgen.1005025

**Published:** 2015-03-16

**Authors:** Yong Ping, Eu-Teum Hahm, Girma Waro, Qian Song, Dai-An Vo-Ba, Ashley Licursi, Han Bao, Logan Ganoe, Kelly Finch, Susan Tsunoda

**Affiliations:** 1 Bio-X Institutes, Key Laboratory for the Genetics of Developmental and Neuropsychiatric Disorders (Ministry of Education), Shanghai Jiao Tong University, Shanghai, China; 2 Department of Biomedical Sciences, Colorado State University, Fort Collins, Colorado, United States of America; University of Florida, UNITED STATES

## Abstract

Alzheimer’s disease (AD) is the most prevalent form of dementia in the elderly. β-amyloid (Aβ) accumulation in the brain is thought to be a primary event leading to eventual cognitive and motor dysfunction in AD. Aβ has been shown to promote neuronal hyperactivity, which is consistent with enhanced seizure activity in mouse models and AD patients. Little, however, is known about whether, and how, increased excitability contributes to downstream pathologies of AD. Here, we show that overexpression of human Aβ42 in a *Drosophila* model indeed induces increased neuronal activity. We found that the underlying mechanism involves the selective degradation of the A-type K+ channel, Kv4. An age-dependent loss of Kv4 leads to an increased probability of AP firing. Interestingly, we find that loss of Kv4 alone results in learning and locomotion defects, as well as a shortened lifespan. To test whether the Aβ42-induced increase in neuronal excitability contributes to, or exacerbates, downstream pathologies, we transgenically over-expressed Kv4 to near wild-type levels in Aβ42-expressing animals. We show that restoration of Kv4 attenuated age-dependent learning and locomotor deficits, slowed the onset of neurodegeneration, and partially rescued premature death seen in Aβ42-expressing animals. We conclude that Aβ42-induced hyperactivity plays a critical role in the age-dependent cognitive and motor decline of this Aβ42-*Drosophila* model, and possibly in AD.

## Author Summary

Alzheimer’s disease (AD) is the most prevalent form of dementia in the elderly population. While it is established that β-amyloid (Aβ) peptide accumulation is a primary even leading to AD, there is little known about how Aβ induces progressive neurodegeneration and decline in cognitive and motor function. Recently, over-production of Aβ has been shown to result in increased neuronal excitability, and Ca^2+^ “overload”, in hippocampal and cortical neurons. Increased excitability is also consistent with behavioral studies which have shown enhanced seizure activity in mouse models with increased Aβ expression, and increased risk of epilepsy in AD patients. We use a transgenic *Drosophila* model that expresses the secreted human Aβ42; this Aβ42-*Drosophila* line exhibits many of the hallmarks of AD. We show that the Aβ42-*Drosophila* line also displays increased neuronal excitability. We determine that the increase in excitability is due to the degradation of a specific K^+^ channel, K_v_4. We then show that genetic restoration of K_v_4 attenuates age-dependent learning and locomotor deficits, slows the onset of neurodegeneration, and partially rescues premature death seen in Aβ42-expressing animals. We conclude that Aβ42-induced hyperactivity plays a critical role in the age-dependent cognitive and motor decline of this Aβ42-*Drosophila* model, and possibly in AD.

## Introduction

The accumulation of β-amyloid (Aβ) oligomers in the brain has strongly been implicated as a primary event in the progression of Alzheimer’s disease (AD) [1–4]. While many studies have shown that Aβ induces excitatory synaptic depression [5,6], more recent reports suggest that Aβ expression also leads to neuronal hyperactivity in cortical and hippocampal neurons [7–13]. Interestingly, increased excitability and Ca^2+^ overload are correlated with neurons in the vicinity of Aβ plaques [9,10]. Much remains to be understood about how Aβ induces both hypo- and hyperactivity, and how these changes are temporally coordinated in the pathogenesis of AD. Some reports suggest that soluble and protofibrillar Aβ induce early hyperactivity that precedes neuronal silencing [7,8,12].

While increased excitability is consistent with enhanced seizure activity in Aβ expressing mouse models [7,11] and increased risk of epilepsy in AD patients [14], it is unknown whether, and to what extent, hyperactivity contributes to downstream effects associated with Aβ accumulation, such as impaired cognitive and motor function. In this study, our intent was to restore excitability to an Aβ-expressing model system and examine whether these age-associated effects would be ameliorated *in vivo*. To do this, it was critical to first identify key mechanism(s) by which excitability is increased by Aβ. A few reports have identified potential contributors to Aβ-induced hyperactivity. For example, neocortical pyramidal cells and dentate granule cells from mice overexpressing Aβ have been observed to have a more depolarized resting potential, resulting in lower action potential thresholds and increased bursts of firing [11]. An increase in spike afterdepolarization has been reported in CA1 pyramidal neurons from transgenic mice overexpressing Aβ [13]. More recently, a transgenic mouse line expressing high levels of human Aβ has been reported to have decreased levels of the voltage-gated Na^+^ channel, Na_v_1.1, in parvalbumin inhibitory interneurons [15].

Here, we use a transgenic *Drosophila* line that over-expresses a secreted form of the toxic human Aβ_1–42_ (Aβ42). These flies exhibit many of the pathogenic hallmarks associated with AD: extracellular amyloid deposits, age-dependent learning and locomotor defects, progressive neurodegeneration and premature death [16–18]. We first show that expression of human Aβ42 indeed induces neuronal hyperexcitability. Since fly brains do not share the same circuitry or complexity of mammalian systems, examining underlying circuit changes would not be meaningful. Intrinsic changes, however, are more likely to be conserved and easier to genetically “reverse”. In addition, while virtually all ion channel families are represented in flies, many families are distilled to a single gene in flies, making intrinsic changes more easily detectable. We characterized Aβ42-induced changes in excitability and show that these changes are explained by selective degradation of the highly conserved A-type K^+^ channel, K_v_4. We then transgenically increased K_v_4 to wild-type levels in Aβ42-expressing animals. We show that when excitability is restored, nearly all Aβ42-induced downstream effects including, neurodegeneration, locomotor and learning defects, and premature death, were attenuated, and in some cases, fully rescued.

## Results

### Characterizing Aβ42-Induced Hyperexcitability

In this study, we used a transgenic line expressing human Aβ42 fused to an N-terminal rat pre-proenkephalin signal peptide that has been shown by two different groups to direct expression, cleavage of the signal sequence, and extracellular secretion of Aβ42 [16,17], thereby mimicking human Aβ42 accumulation in the extracellular space around neurons. This sequence was placed under the control of an *upstream activating sequence* (*UAS*), *UAS-Aβ42* [18]). Using the *UAS-GAL4* system in *Drosophila* [19], *UAS-*Aβ42 expression was activated with the pan neuronal driver *elav-GAL4* (*elav-GAL4;UAS-Aβ42/+*). We first examined if these neurons from Aβ42-secreting cultures displayed increased excitability. We performed current-clamp recordings from primary neurons that were dissociated from late-gastrula stage embryos and grown for up to two weeks in culture. These neurons likely represent larval stage neurons and are among the best characterized neurons in *Drosophila* with respect to voltage-dependent channels and synaptic physiology [20–26].

**Fig 1:**
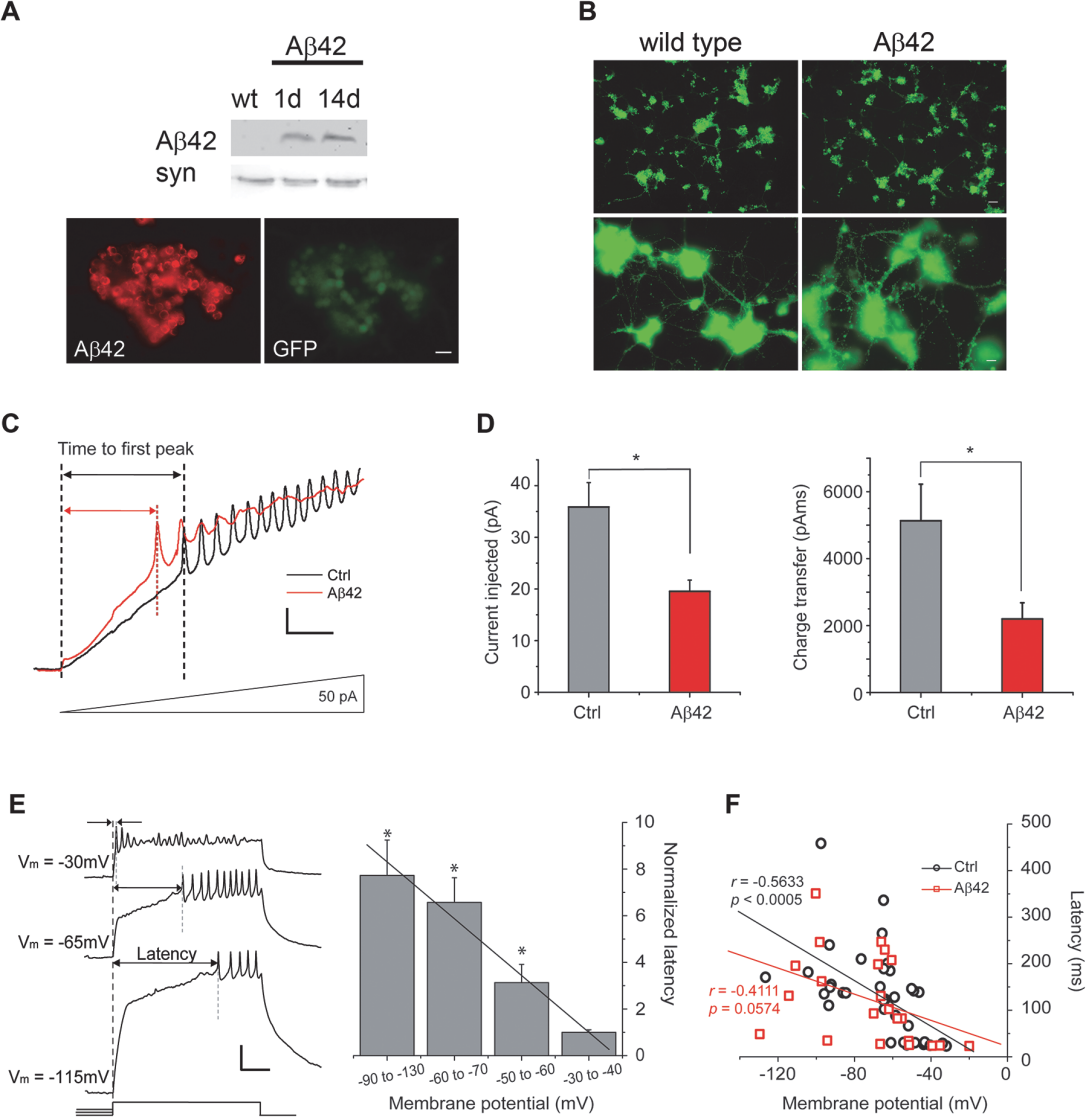
Aβ42 induces increased neuronal excitability in cultures 9 days old. (A) Representative immunoblots of fly heads (*Top*) and immunostaining of cultured neurons (*Bottom*) showing expression of Aβ42 in *elav-GAL4;UAS-Aβ42/+* (Aβ42) heads and *elav-GAL4;UAS-Aβ42/+;UAS-GFP* neurons, respectively; absence of Aβ42 expression in wild-type (wt) is shown as a negative control. Note that immunostaining is from a large cluster of neurons identified by GFP expression. Scale bar represents 12.5 μm. (B) Shown are representative wild-type and *elav-GAL4;UAS-Aβ42/+* (Aβ42) cell cultures, both with neurons expressing GFP as a marker, at 9 days. At lower power (*Top*), whole cultures from a single-embryo can be seen (scale bar represents 25 μm); at higher power (*Bottom*), neuronal processes between clusters of neurons can be seen (scale bar represents 8 μm). No obvious differences in growth of cultured neurons between wt and Aβ42 were observed. (C) Representative current-clamp recordings from wild-type (Ctrl) and *elav-GAL4;UAS-Aβ42/+* (Aβ42) neurons, showing relative times to the first action potential (AP) firing in response to ramped current stimulation. Current injection was ramped from 0 to 150 pA over 1 sec; voltage responses to the first 336 ms (50 pA) of the ramp protocol are shown. Scale bars represent 50 ms and 10 mV. (D) Quantification of the injected current (*Left*) and the charge transfer (*Right*) required to elicit the first AP in Ctrl and Aβ42 neurons during ramp stimulation; all ramp protocols were initiated from a membrane potential of -50 to -60 mV. The average charge transfer to elicit AP firing was also significantly reduced in Aβ42 neurons (2197.4 +/- 484.46 pAms, n = 6), compared to Ctrl (5132.8 +/- 1091.32 pAms, n = 13; *P* < 0.05, Student’s t-test). (E) Representative traces (*Left*) from a wild-type neuron, showing the latency to AP firing in response to a 500 ms pulse of 20 pA current injection, following pre-injections of current that generated a membrane potential of -115, -65, or -30 mV. Latencies to AP firing was normalized to average latency at -30 to -40 mV are shown for wild-type neurons from the indicated membrane potentials (*Right*). Note the negative correlation between AP latency and membrane potential (* denotes a significant difference in latency compared to that at V_m_ = -30 to -40mV; *P* < 0.05, Student’s t-test). Scale bar represents 100 ms and 20 mV. (F) AP latencies, in response to 500 ms, 40 pA current pulses, from single events are plotted against membrane potential for Ctrl and Aβ42 neurons. Ctrl cells showed a significant correlation between latency and membrane potential (*r* = -0.5633, *P* < 0.0005, n = 37), while Aβ42 cells did not (*r* = -0.4111, *P* = 0.0574, n = 22); the coefficient of correlation for each genotype was calculated by the Pearson product-moment correlation coefficients (see Materials and Methods).

We grew cultures in 20 μL drops of media and aged them for 9 days to allow for Aβ42 expression, secretion, and accumulation; Aβ42 expression/accumulation was confirmed by immunoblot analyses (Fig. 1A) and immunostaining of cultures (Fig. 1B). No differences in neuronal growth could be seen between Aβ42-expressing cultures and control cultures (Fig. 1B). We examined action potential (AP) firing in these neurons response to an injection of current ramped from 0 to 150 pA over a 1-second period; pre-injection of current was applied to normalize membrane potentials to -50 to -60 mV (similar to the average resting potential of these cells) before ramped stimulation was applied. We found that neurons from Aβ42-expressing/secreting cultures (also referred to as Aβ42 neurons) consistently fired more readily, with less current injection, compared to neurons from a genetic background control line (Fig. 1A). AP firing in Aβ42 neurons was initiated when ramp stimulation reached only 19.5 +/- 2.2 pA (n = 6; Fig. 1B); in contrast, control neurons fired when current injection reached 35.9 +/- 4.7 pA (n = 13; Fig. 1B). The charge transfer required for AP firing in Aβ42 neurons was less than half of what was needed for wild-type neurons (Fig. 1B). These results suggest that neurons from Aβ42-expressing cultures indeed exhibit increased susceptibility to firing.

To further characterize the hyperactivity of Aβ42 neurons, we compared AP firing in response to 500 ms current injections in neurons from background control lines and neurons from Aβ42-expressing cultures. Current pulses of 40 pA were used to examine the latency to the first AP firing; for neurons that did not fire APs, we increased current injections by 20 pA until APs were elicited. Wild-type neurons displayed a clear negative correlation between membrane potential and latency to the first AP firing (Fig. 1C-D). Interestingly, this latency to the first AP is dependent almost exclusively on K_v_4 channels [26], which carry an A-type K^+^ current that is activated at subthreshold potentials. The number of K_v_4 channels available for activation is dependent on membrane potential, such that the more depolarized the membrane potential, the smaller the number of K_v_4 channels available for activation [21] and therefore, the shorter the latency to AP firing. Neurons from Aβ42-expressing cultures, in contrast to wild-type, displayed significantly reduced correlation between membrane potential and latency to AP firing (Fig. 1D). These results suggested that K_v_4 channels, in particular, may be impacted by Aβ42 expression.

### K_v_4 Current Is Selectively Decreased by Aβ42 Expression

To test the hypothesis that K_v_4 channels are affected by Aβ42 expression, we performed voltage-clamp studies to examine whether K_v_4, or any other voltage-dependent K^+^ currents, were altered in Aβ42-expressing cultures. Fortunately, previous studies have genetically identified all of the voltage-dependent K^+^ currents present in these neurons: all of the A-type K^+^ currents (I_A_) present in the cell bodies of these neurons are carried by channels encoded by the single *K*_*v*_ *4* (*Shal*) gene in *Drosophila*, while the delayed rectifier (DR) component has been shown to be encoded largely by the single *K*_*v*_ *2* (*Shab*) gene, and in small part by the *K*_*v*_ *3* (*Shaw*) gene [20,21]. The K_v_2-K_v_3 DR component was recorded using a prepulse of -45 mV to completely inactivate K_v_4 channels, before stepping to a depolarized test potential; the K_v_4 current was then isolated by subtracting the DR component from the whole-cell current, as performed in previous studies [20,21,25,26]. Since Aβ42 is thought to have especially detrimental effects for neurons involved in cognitive function, we focused on Kenyon cells of the mushroom bodies (MBs), which have been shown to constitute the major site of olfactory learning and memory formation in the fly brain [27–30]. To identify MB neurons, we crossed transgenic *elav-GAL4;UAS-Aβ42/+* flies to flies containing a *GFP*.*S65T*.*T10* transgene, which has been shown to drive GFP expression only in MB neurons, regardless of the *GAL4* driver present [30,31].

**Fig 2:**
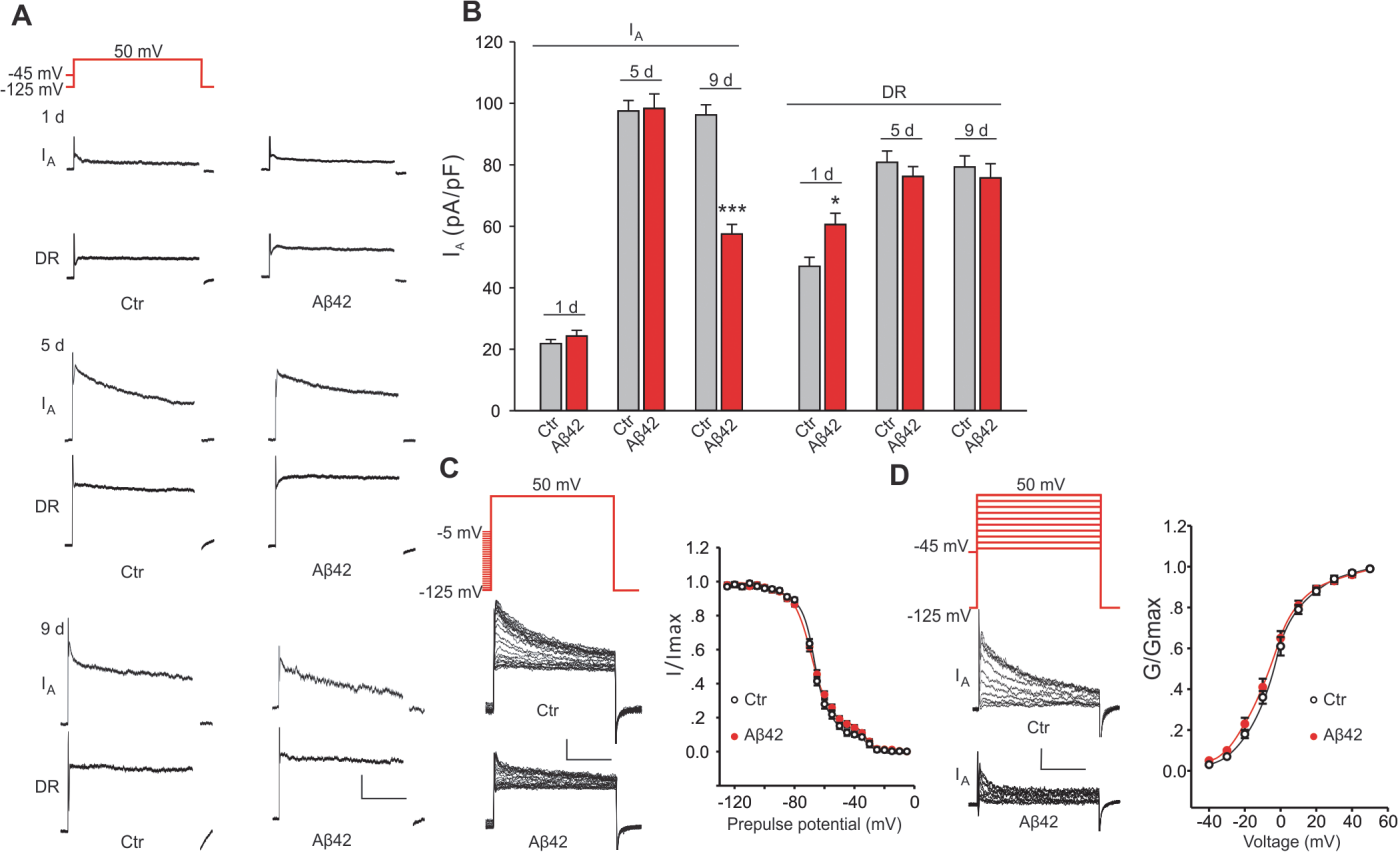
Aβ42-induced changes in voltage-dependent K^+^ currents in primary neurons. Representative K^+^ current traces (A) and quantification of current amplitudes (B) from MB neuron recordings from *UAS-Aβ42/+* (Ctr) and *elav-GAL4;UAS-Aβ42/+* (Aβ42) embryos cultured for 1, 5 and 9 days. The K_v_2-K_v_3 DR component was recorded using a prepulse of -45 mV to completely inactivate K_v_4 channels, before stepping to a test potential of +50 mV; the K_v_4 current was then isolated by subtracting this DR component from the total whole-cell current elicited from a prepulse of -125 mV, stepping to a test potential of +50 mV. Current density was obtained by dividing peak current amplitude by cell capacitance. K_v_4 current density was significantly reduced in Aβ42 MB neurons in 9-day old cultures, while no difference in K_v_2-K_v_3 currents was observed. (n = 8–10 for each group, * *P* < 0.05, ** *P* < 0.01, Student’s t-test). Scale bars, 100 pA, 50 ms. (C-D) Steady-state inactivation and activation properties of Kv4 compared between neurons from Ctr and Aβ42 cultures. For steady-state inactivation analyses, we used a prepulse from -125 to -5 mV, in 5 mV intervals, then stepped to a test potential of +50 mV; representative current traces are shown (C, *Left*). For G –V analyses, we used voltage jumps from -40 to 50 mV, in 10 mV intervals, and either a prepulse of -125 mV or -45 mV for the total current or DR component, respectively; K_v_4 current amplitudes were obtained by subtracting the DR component from the total current. Steady-state inactivation and activation curves were fitted with Boltzmann functions, I/I_max_ = 1/(1 + exp[(V – V_1/2_)/k]) and G/G_max_ = 1/(1 + exp[(V_1/2_ – V)/k]), respectively. V_1/2_ is the half-maximal voltage, and k is the slope factor. Note that steady-state inactivation was fitted by two Boltzmann equations, and K_v_4 is represented by the first Boltzmann (more negative operating range)[20,21]. There was no significant difference in half-maximal inactivation potential values (Ctr, V_1/2_ = -69.3 ± 2.1 mV, k = 9.1 ± 0.5; Aβ42, V_1/2_ = -71.2 ±3.4 mV, k = 9.7 ± 0.5). There was also no difference in the half-maximal activation potential values (Ctr, V_1/2_ = -5.7 ± 0.4 mV, k = 12.5 ± 0.8; Aβ42, V_1/2_ = -6.8 ± 0.4 mV, k = 13.6 ± 0.6). n = 10 or each group. *** P < 0.001. All neurons were from cultures 9 days old.

We recorded from GFP-labeled MB neurons from 20 μL drop cultures at 1, 5, and 9 days. We found that the K_v_2-K_v_3 DR component was increased in Aβ42 neurons at 1 day, compared to control neurons from relevant genetic background lines (*UAS-Aβ42*/+ or *elav-GAL4*); this increase, however, was not persistent after the first day (Figs. 2 and S1). More prominent was a 37% decrease in the K_v_4 current density that developed in Aβ42 neurons after 9 days compared to controls (Figs. 2 and S1); no differences were seen in cell capacitance (Ctrl: 2.7 +/- 0.2 pF, Aβ42: 2.5 +/- 0.1 pF), resting membrane potential (Ctrl: -55.0 +/- 2.3 mV, Aβ42: -52.1 +/- 2.6 mV), or input resistance (Ctrl: 2.1 +/- 0.2 GΩ, Aβ42: 2.2 +/- 0.2 GΩ). The loss of K_v_4 current is consistent with current-clamp recordings that show a loss of correlation between membrane potential and latency to AP firing (Fig. 1D).

We also examined whether the apparent loss of K_v_4 current in Aβ42-expressing neurons might be due to an Aβ42-induced change in K_v_4 inactivation or activation properties. We compared steady-state inactivation properties of K_v_4 currents in Aβ42-expressing neurons and background controls. Whole-cell currents inactivated with more depolarized pre-pulses. As previously characterized, this inactivation was best fit with a double Boltzman equation with the component that inactivates at more hyperpolarized potentials corresponding to the K_v_4 current [20,21]. No differences were observed between Aβ42-expressing and background contols (Fig. 2C). Activation properties were also not altered by Aβ42 expression (Fig. 2D). These results suggest that the Aβ42-induced decrease in K_v_4 current density is not due to a change in steady-state inactivation or activation properties of the channel.

**Fig 3:**
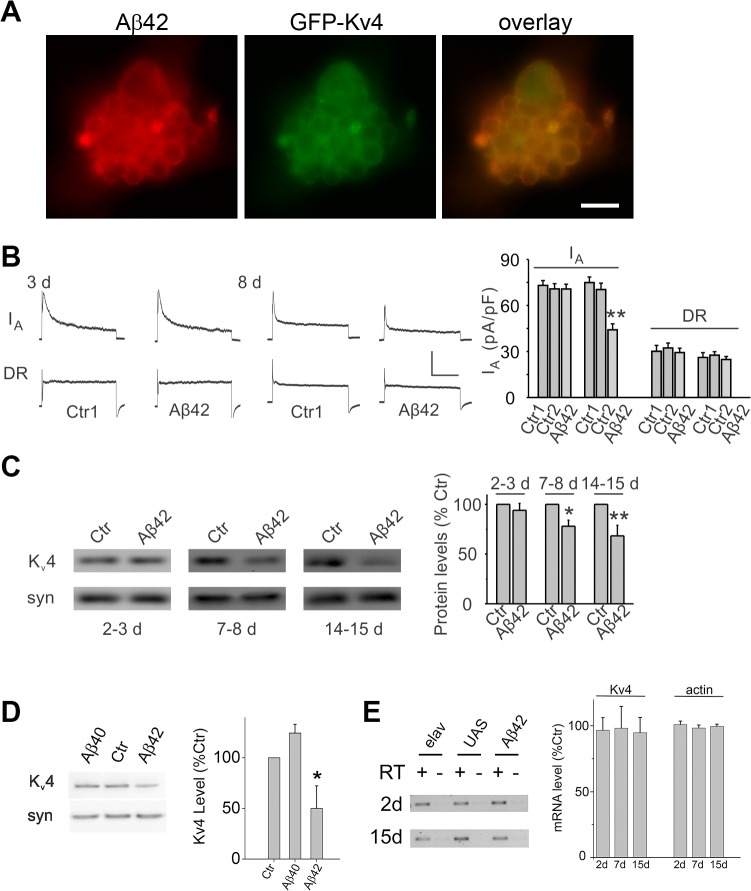
Expression of Aβ42 decreases K_v_4 protein and currents in the intact brain. (A) Representative neuronal cluster from 9 day old culture from *elav-GAL4;UAS-Aβ42/UAS-GFP-K*_*v*_ *4* embryos show co-localization of Aβ42 (red) and GFP-K_v_4 (green) immunostaining. Scale bar represents 8.3 μm. (B) Representative K_v_4 and K_v_2-K_v_3 traces (*Left*), and K_v_4 current density quantification (*Right*) from GFP-labeled MB neurons in *UAS-Aβ42/+* (Ctr) and *elav-GAL4;UAS-Aβ42/+* (Aβ42) intact brains at 3 and 8 days; Ctr1 and Ctr2 on right correspond to *UAS-Aβ42/+* and *elav-GAL4;+*, respectively. The K_v_2-K_v_3 DR component was recorded using a prepulse of -45 mV to completely inactivate K_v_4 channels, before stepping to a test potential of +50 mV; the K_v_4 current was then isolated by subtracting this DR component from the total whole-cell current elicited from a prepulse of -125 mV, stepping to a test potential of +50 mV. Current density was obtained by dividing peak current amplitude by cell capacitance. K_v_4 current density in Aβ42 neurons at 8 days were significantly reduced compared to Ctr1 and Ctr2 neurons (*P* < 0.05, n = 6–13 for each genotype). Scale bars represent 50 pA and 50 ms. (C) Representative immunoblots (*Left*) and quantitative analyses (*Right*) of K_v_4 protein levels in *UAS-Aβ42/+* (Ctr) and *elav-GAL4;UAS-Aβ42/+* (Aβ42) heads at 2–3, 7–8, and 14–15 days after eclosion. Protein levels were normalized to the loading control signal from anti-syntaxin (syn) (n = 4 for each group, * *P* < 0.05, ** *P* < 0.01, Student’s t-test). (D) Representative immunoblots (*Left*) and quantitative analyses (*Right*) of K_v_4 protein from *elav* (Ctr), *elav;;UAS-Aβ40/+* (Aβ40), and *elav;UAS-Aβ42/+* heads at 14–15 days AE. (n = 3–4 for each group, * *P* < 0.05, Student’s t-test indicates significant difference between Aβ40 and Aβ42) (E) Representative blots (*Left*) and quantitative results (*Right*) from RT-PCR analyses for *K*_*v*_ *4* and *actin* RNA levels from *elav* (Ctr), *UAS-Aβ42* (UAS), and *elav;UAS-Aβ42/+* (Aβ42) flies aged 2, 7, and 15 days AE. Quantitation was performed from RT-PCR results from three independent RNA extractions; RT minus controls were always performed in parallel with RT plus reactions as indicated. No significant differences were seen across the three genotypes.

To examine if Aβ42 accumulations seen in these cultures might co-localize with K_v_4 channels, we made cultures from *elav-GAL4;UAS-Aβ42/UAS-GFP-K*_*v*_ *4* animals and performed co-immunostaining using antibodies against Aβ42 and GFP. Interestingly, after 9 days in culture, Aβ42 seemed to accumulate especially near the membranes of neurons. Fig. 3A shows anti-Aβ42 staining overlapping with GFP-K_v_4 labeling, suggesting a direct interaction.

We next examined whether a reduction in the K_v_4 current would also be observed in the intact adult brain. Background control lines and transgenic *elav-GAL4;UAS-Aβ42/+* flies were aged for 3 or 8 days after eclosion (AE); whole brains were acutely dissected; MB neurons were GFP labeled as described above. No difference in K^+^ current density was observed at 3 days. At 8 days, however, we found that K_v_4 current density was significantly reduced in MB neurons from Aβ42-expressing brains (Fig. 3B). No differences in cell capacitance (Ctrl: 1.9 +/-0.1 pF, Aβ42: 1.8 +/- 0.1 pF), resting membrane potential (Ctrl: -52.8 +/- 2.3 mV, Aβ42: -49.1 +/- 1.7 mV), or input resistance (Ctrl: 2.5 +/- 0.1 GΩ, Aβ42: 2.4 +/- 0.2 GΩ) were observed between control and Aβ42 neurons. Our results in primary cultures and the intact brain both indicate that the K_v_4 current, and not the K_v_2-K_v_3 DR current, is affected by Aβ42 with age.

### Aβ42 Induces Degradation of K_v_4 Protein via a Pathway Dependent on Both the Proteasome and Lysosome

To determine if the reduction in K_v_4 current is due to a decrease in channel protein, we performed immunoblot analyses on heads from an *elav-GAL4;UAS-Aβ42/+* flies compared to background control lines. Steady-state levels of K_v_4 protein were not significantly different at 2–3 days AE. At 7–8 days, however, heads from *elav-GAL4;UAS-Aβ42/+* flies displayed a decrease in K_v_4 protein, compared to controls; this decrease continued to persist for at least 14–15 days AE (Figs. 3C and S2). To test whether the decrease in K_v_4 was specific to the expression of Aβ42, we also examined a similar transgenic line generated to over-express a secreted human Aβ40 peptide [16]. We found that steady-state K_v_4 protein levels in *elav-GAL4;;UAS-Aβ40/+* fly heads were not significantly different from background control lines at 14–15 days AE, in contrast to *elav-GAL4;UAS-Aβ42/+* heads (Fig. 3D). These results suggest that Aβ42, but not Aβ40, induces an age-dependent loss of K_v_4.

To investigate the mechanism by which K_v_4 protein loss occurs in *elav-GAL4;UAS-Aβ42/+* flies, we first examined whether mRNA levels of K_v_4 were altered by Aβ42 expression. We isolated RNA from *elav-GAL4;UAS-Aβ42/+* and background controls, *elav;+* and *UAS-Aβ42/+*, then performed RT-PCR using primers for K_v_4. These experiments were performed using flies that were aged 2 days, 7 days, and 15 days AE; RNA extractions from three different pools of aged flies were used. We found no significant difference in *K*_*v*_ *4* mRNA levels from 2 to 15 days (Fig. 3E), suggesting that the reduction in K_v_4 is not likely to be due to a change in transcript level.

**Fig 4:**
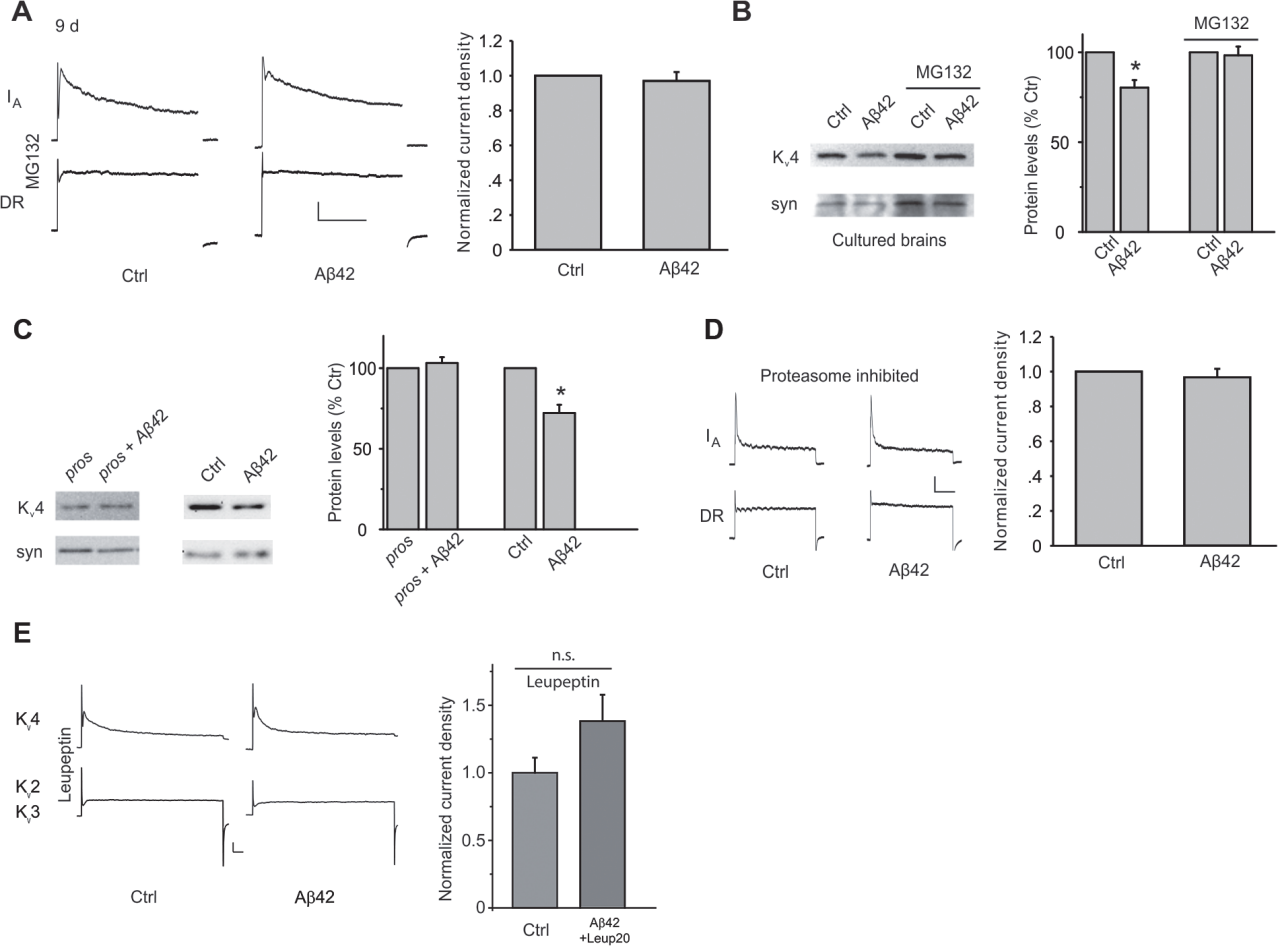
Aβ42-induced down-regulation of K_v_4 protein is mediated by a proteasome and lysosome-dependent pathway. (A) Representative traces of separated K_v_4 and K_v_2-K_v_3 currents recorded from MB neurons from *UAS-Aβ42/+* (Ctr) and *elav-GAL4;UAS-Aβ42/+* (Aβ42) cultures at 9 days, after incubation with MG132 (10 μM). The K_v_2-K_v_3 DR component was recorded using a prepulse of -45 mV to completely inactivate K_v_4 channels, before stepping to a test potential of +50 mV; the K_v_4 current was then isolated by subtracting this DR component from the total whole-cell current elicited from a prepulse of -125 mV, stepping to a test potential of +50 mV. Current density was obtained by dividing peak current amplitude by cell capacitance. Quantitative analyses of K_v_4 current density shows that MG132 blocked the Aβ42-induced down-regulation of K_v_4 (*Right;* n = 8–9 for each group, ** *P* < 0.01, Student’s t-test). Scale bars represent 100 pA and 50 ms. (B) Representative immunoblots and quantitative analyses of K_v_4 protein levels in cultured brains. The Aβ42-induced decrease in K_v_4 is blocked when brains were incubated with MG132 for 4 days. K_v_4 levels were normalized to the loading control signal from anti-syntaxin (syn) (n = 3 for each group, * *P* < 0.05, Student’s t-test). (C) Down-regulation of K_v_4 protein by Aβ42 is absent when proteasome activity was inhibited by expression of the dominant temperature-sensitive *UAS-Pros26* ^*1*^ *and UASProsβ* ^*2*^ transgenes. *elav-GAL4;UAS-pros26* ^*1*^/+;*UAS-prosβ* ^*2*^/+ flies (pros), *elav-GAL4;UAS-Aβ42/UAS-pros26* ^*1*^;*UAS-prosβ* ^*2*^/+ flies (pros + Aβ42), *UAS-Aβ42/+* (Ctr), and *elav-GAL4;UAS-Aβ42/+* (Aβ42) were all raised at 18°C during development, then shifted to 29°C for 7–8 days after adult eclosion. Immunoblot analyses were performed for K_v_4, and normalized to syn (*Left*). For quantitative analyses (*Right*), n = 4 for each condition, * *P* < 0.05, Student’s t-test. (D) Aβ42-induced decrease in K_v_4 current is also absent by the genetic inhibition of the proteasome described in (C) (*right*). *elav-GAL4;UAS-Pros26* ^*1*^/*UAS-Aβ42;UAS-Prosβ* ^*2*^/+ and *elav-GAL4;UAS-Pros26* ^*1*^/+;*UAS-Prosβ* ^*2*^/+ flies were raised at 18°C during development, then shifted as newly-eclosed adult flies to 29°C for 7–8 days (n = 4 for immunoblots, and n = 8 for each current recordings). Scale bars represent 25 pA and 50 ms. (E) Representative K_v_4 and K_v_2–3 currents in Ctrl and *elav-GAL4;UAS-Aβ42* (Aβ42) neurons from cultures treated with 20 μM leupeptin for 8 days. 20 μM leupeptin blocked the Aβ42-induced decrease in K_v_4 current density seen in Ctrl neurons.

We next tested the hypothesis that Aβ42 induces an increase in K_v_4 protein degradation. Since major degradation pathways include the proteasome and/or lysosome, we examined whether Aβ42-induced loss of K_v_4 required function of either. To test the possible involvement of the proteasome, neurons dissociated from *elav-GAL4;UAS-Aβ42/+* and *UAS-Aβ42/+* lines were cultured for 9 days in the presence or absence of MG132, which is known to block proteasome function. We found that MG132 completely blocked the Aβ42-induced decrease in I_A_ (Fig. 4A), suggesting that Aβ42 induces degradation of K_v_4 channels via a proteasome-dependent pathway. To test the proteasome’s involvement *in situ*, we dissected brains at 2 days AE, then maintained them in culture for an additional 4–5 days, as previously performed [25]. Dissected *elav-GAL4;UAS-Aβ42/+* and *UAS-Aβ42/+* brains were mock and MG132-treated for 4 days. Immunoblot analyses showed that Aβ42 still induced a 22% decrease in K_v_4 protein in cultured brains (Fig. 4B). The decrease, however, was completely blocked in the presence of MG132 (Fig. 4B). We also tested the involvement of the proteasome *in vivo*, using transgenic lines that over-express *Pros26* ^*1*^ and *Prosβ* ^*2*^, dominant-negative mutant forms of the 20S proteasome subunits β6 and β2, respectively [32]. Flies expressing *Pros26* ^*1*^ and/or *Prosβ* ^*2*^ alleles have been shown to exhibit normal proteasome function at 18°C, but severely impaired function when shifted to 29°C [33,34]. We raised *elav-GAL4;UAS-Pros26* ^*1*^/*UAS-Aβ42;UAS-Prosβ* ^*2*^/+ and *elav-GAL4;UAS-Pros26* ^*1*^/+;*UAS-Prosβ* ^*2*^/+ flies at 18°C during development, then shifted newly-eclosed adult flies to 29°C for 7–8 days. We found that when proteasome activity was inhibited, Aβ42 did not induce a decrease in K_v_4 protein, as assayed by immunoblot analyses, or a decrease in the K_v_4 current, as recorded from MB neurons in the intact brain (Fig. 4C-D). To ensure that an Aβ42-induced decrease in K_v_4 could still be expected with the temperature-shift protocol used, we performed similar experiments with *elav-GAL4;UAS-Aβ42/+* and *UAS-Aβ42/+*. Aβ42 expression still induced a decrease of K_v_4 protein after a temperature shift to 29°C (Fig. 4C). Together, these results strongly suggest that Aβ42 induces a loss of K_v_4 protein by way of a proteasome-dependent pathway.

Since turnover of other ion channels and receptors have been shown to be dependent on both the proteasome and lysosome[35–38], we also tested for the possible involvement of the lysosome. We used leupeptin, which is known to block lysosomal function, in wild-type and Aβ42-expressing neuronal cultures. One day after cell cultures were established, leupeptin was added, and cultures were maintained for another 8 days before recording K_v_4 currents. We found that leupeptin, at 20 μM, blocked the decrease in K_v_4 current in Aβ42-expressing neurons (Fig. 4E). With 10 μM leupeptin, however, K_v_4 currents were still decreased in Aβ42-neurons (Fig. 4E), indicating a concentration dependent block of the decrease in K_v_4 current seen in Aβ42-neurons. These results suggest that the lysosome, in addition to the proteasome, is involved in Aβ42-induced K_v_4 degradation.

### Transgenic Restoration of K_v_4 Rescues Hyperexcitability

**Fig 5:**
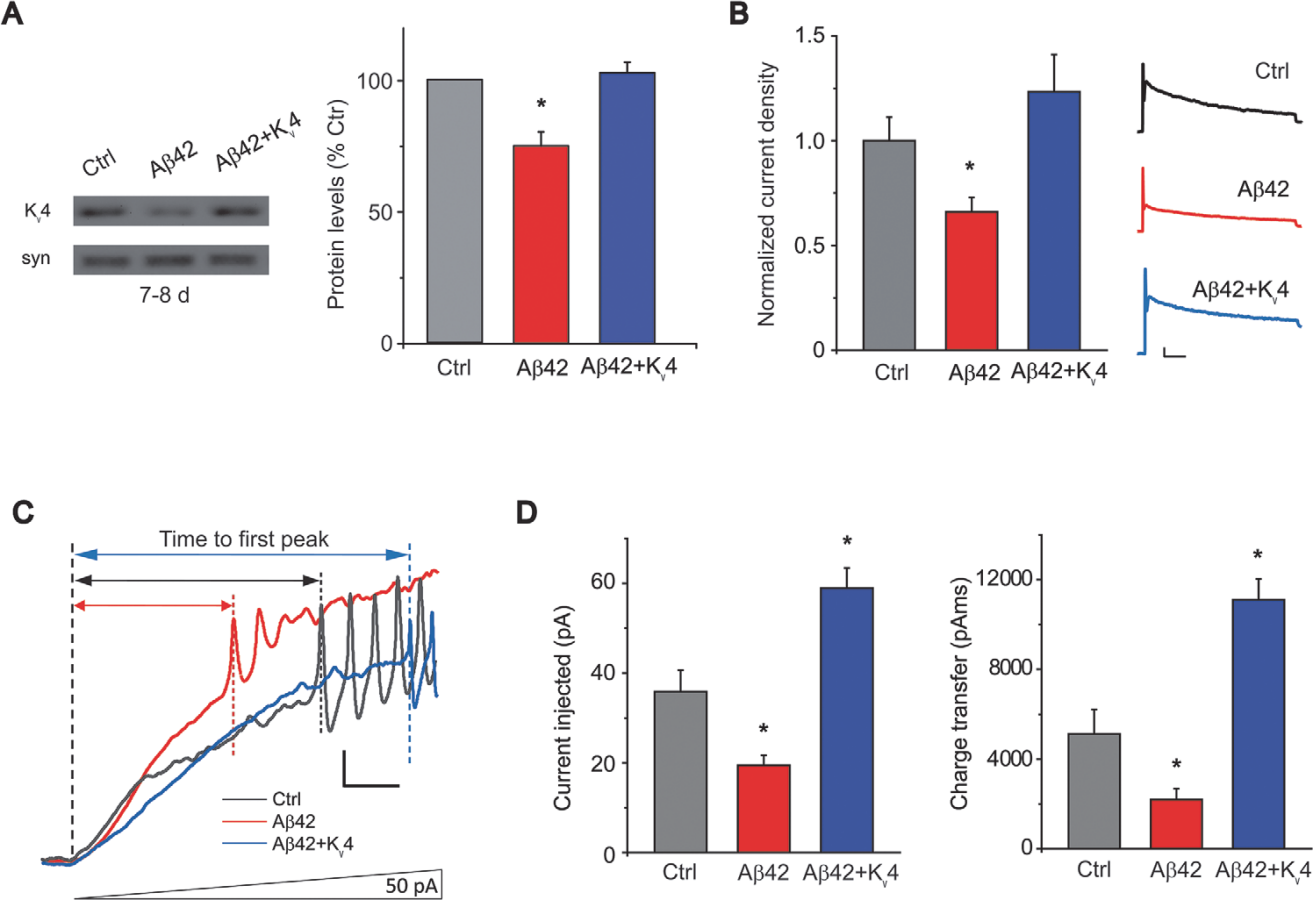
Transgenic over-expression of K_v_4 in Aβ42-expressing flies restores K_v_4 protein levels and normal excitability to neurons. (A) Immunoblot analyses of *UAS-Aβ42/+* (Ctrl), *elav-GAL4;UAS-Aβ42/+* (Aβ42), and *elav-GAL4;UAS-Aβ42/UAS-K*_*v*_ *4* (Aβ42 + K_v_4) fly heads at 7–8 days are shown. Transgenic expression of *UAS-K*_*v*_ *4* restores K_v_4 protein levels to wild type levels. (n = 3 for each group, * *P* < 0.05, Student’s t test). (B) Representative K_v_4 currents isolated in voltage-clamp mode from primary neurons, from cultures 9 days old, from Ctrl, Aβ42 and *elav-GAL4;UASAβ42/UAS-K*_*v*_ *4* (Aβ42+K_v_4) flies are shown (*Right*). The K_v_2-K_v_3 DR component was recorded using a prepulse of -45 mV to completely inactivate K_v_4 channels, before stepping to a test potential of +50 mV; the K_v_4 current was then isolated by subtracting this DR component from the total whole-cell current elicited from a prepulse of -125 mV, stepping to a test potential of +50 mV. Current density was obtained by dividing peak current amplitude by cell capacitance. Quantitative analyses (*Left*) show that K_v_4 current density from Aβ42 neurons (194.3 +/- 23.68 pA, *n* = 9) were significantly smaller than from Ctrl neurons (320.2 +/- 33.52 pA, *n* = 25). K_v_4 current density in Aβ42+K_v_4 neurons showed a near complete restoration (334.3 ± 48.97 pA, n = 18). * *P* < 0.05, Student’s *t*-test. Scale bars represent 100 pA and 20 ms. (C) Representative current-clamp recordings from Aβ42 and the Aβ42+K_v_4 neurons in response to ramped stimulation protocol described in Fig. 1A, show that the time to first peak was significantly prolonged by the restoration of K_v_4 to Aβ42-expressing neurons. Scale bar represents 10 mV and 50 ms. (D) Quantification of the average current injected and charge transfer required to elicit the first AP are shown. Both were significantly increased with transgenic restoration of K_v_4: 58.9 +/- 4.54 pA, *n* = 9 (*Left*), and 11,108.0 +/- 920.49 pAms, *n* = 9 (*Right*); note that quantification for Ctrl and Aβ42 values represent the same data set shown in Fig. 1B. * *P* < 0.05, Student’s *t*-test.

To test whether we could restore Aβ42-induced hyperexcitability, we generated transgenic lines over-expressing *UAS-K*_*v*_ *4* in Aβ42-expressing animals. More than 10 *UAS-K*_*v*_ *4* lines were isolated with different insertion sites of the transgene; each line was crossed to an *elav-GAL4* driver and expression levels were tested by immunoblot analysis. We selected lines that raised K_v_4 protein levels and K_v_4 current amplitudes in Aβ42-expressing heads to near wild-type levels (Fig. 5A-B); we refer to these as Aβ42+K_v_4 neurons (*elav-GAL4;UAS-Aβ42/UAS-K*_*v*_ *4*). We then performed current-clamp recordings to determine if restoration of K_v_4 reduced excitability in ramp stimulation protocols, as performed in Fig. 1A. We found that in Aβ42+K_v_4 neurons, the average current (and charge transfer) required to induce AP firing was significantly increased, compared to neurons from cultures expressing Aβ42 alone, and was quite similar to background control neurons (Fig. 5C-D). These results suggest that the decreased threshold for AP firing observed in Aβ42 neurons is indeed due to the loss of K_v_4 channels. We then used these Aβ42+K_v_4 lines to test whether downstream cognitive and motor dysfunction would be attenuated.

### Loss of K_v_4 Plays a Critical Role in Aβ42-Induced Learning Defects

We first investigated whether Aβ42-induced loss of K_v_4 would be predicted to affect learning in this fly model. We reasoned that the Aβ42-induced changes observed in our aged late-embryonic cultures were likely to be relevant at larval stages. So, to examine learning, we used an odor-association learning assay with third instar larvae, similar to previously published studies [39]. Larvae were trained to associate a neutral odor (amyl acetate (AM), or 1-octanol (OCT)) with fructose, a natural appetitive attractant. In AM+/OCT- training, larvae were trained to associate AM with fructose, while in AM-/OCT+ training, larvae were trained to associate OCT with fructose (see Materials and Methods). After training, larvae were placed on a test plate with an AM source on one side, and an OCT source on the other side; after 20 minutes, the number of larvae on each half of the plate were counted. Standard preference scores (Pref) for AM and OCT were used to calculate a learning index (LI) for each independent pair of groups that had undergone AM+/OCT- and AM/OCT+ training (see Materials and Methods). We performed two sets of studies: first, to examine whether the loss of K_v_4 function alone could lead to learning impairment, and second, to examine the effect of Aβ42 on learning, and to determine if defects would be attenuated by K_v_4 expression. Prior to these studies, we first confirmed that all genotypes showed a naïve preference for fructose (S3A Fig.), no preference for AM or OCT (S3B Fig.), and normal chemotaxis towards known attractants (S3C Fig.).

**Fig 6:**
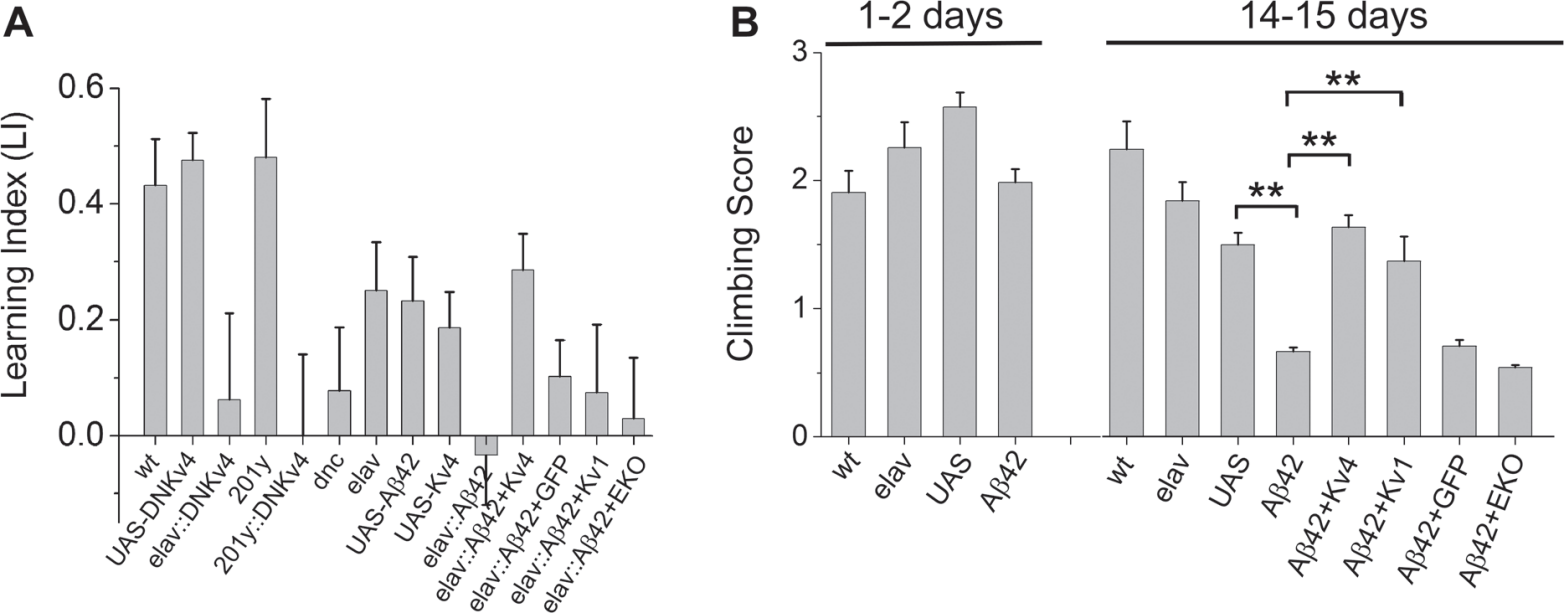
Larval olfactory associative learning and locomotion is defective in Aβ42-expressing larvae, and rescued by K_v_4. (A) The following genotypes were trained and tested for learning function: wild-type (wt), *UAS-DNK*_*v*_ *4* (UAS-DNK_v_4), *elav-GAL4;;UAS-DNK*_*v*_ *4* (elav-GAL4::DNK_v_4), *elav-GAL4* (elav), *UAS-Aβ42/+* (UAS-Aβ42), *UAS-K*_*v*_ *4* (UAS-K_v_4), *elav-GAL4;UAS-Aβ42/+* (elav::Aβ42), *elav-GAL4;UAS-Aβ42/UAS-K*_*v*_ *4* (elav::Aβ42+K_v_4), *elav-GAL4;UAS-Aβ42/UAS-K*_*v*_ *1* (elav::Aβ42+K_v1_), *elav-GAL4;UAS-Aβ42/+;UAS-CD8-GFP* (elav::Aβ42+GFP), *elav-GAL4;UAS-Aβ42/+;UAS-EKO/+* (elav::Aβ42+EKO), *dnc* ^*1*^ (dnc), *201y-GAL4* (201y), and *201y-GAL4;UAS-DNK*_*v*_ *4* (201y::DNK_v_4). Learning was tested by training larvae to associate AM or OCT with fructose in the AM+/OCT- or AM-/OCT+ paradigms described in the text and Materials and Methods. After training, larvae were placed on a plain agarose test plate, with AM and OCT sources at opposite sides; after 20 minutes, larvae on each half of the plate were counted. Preference scores and a learning index (LI) were calculated, as described in Materials and Methods (n = 4–15 pairs of groups for each genotype, with one pair consisting of one group for AM+/OCT- training and one group for AM-/OCT+ training; 5 larvae per individual group). *elav-GAL4;UAS-DNK*_*v*_ *4*, *201y-GAL4;UAS-DNK*_*v*_ *4*, and *dnc* all showed significantly reduced learning indices, compared to wild-type and background controls. *elav-GAL4;UAS-Aβ42/+* also had a significantly reduced LI compared to background controls, which was rescued in *elav-GAL4;UAS-Aβ42/UAS-K*_*v*_ *4*, but not in *elav-GAL4;UAS-Aβ42/+;UAS-CD8-GFP/+*, *elav-GAL4;UAS-Aβ42/UAS-K*_*v*_ *1*, or *elav-GAL4;UAS-Aβ42/+;UAS-EKO/+*. (B) Locomotor activity was tested in a standard climbing assay on wild-type (wt), *elav-GAL4* (elav), *UAS-Aβ42/+* (UAS), *elav-GAL4;UAS-Aβ42/+* (Aβ42), *elav-GAL4;UAS-Aβ42/UAS-K*_*v*_ *4 (*Aβ42+K_v_4), *elav-GAL4;UAS-Aβ42/UAS-K*_*v*_ *1* (Aβ42+K_v1_), *elav-GAL4;UAS-Aβ42/+;UAS-CD8-GFP/+* (Aβ42+GFP), and *elav-GAL4;UAS-Aβ42/+;UAS-EKO/+ (*Aβ42+EKO) at 1–2 days and 14–15 days after eclosion, as described in text; each fly was given one point for every two tubes they climbed out of. The mean score of flies from each group (30–35 flies) was calculated; this was then repeated for 5–15 groups for each genotype with averages shown. *elav-GAL4;UAS-Aβ42/+* flies showed a significant impairment at 14–15 days, compared with *UAS-Aβ42/+* background controls. Co-expression of *UAS-K*_*v*_ *4* or *UAS-K*_*v*_ *1* rescues this impairment, while expression of *UAS-EKO* did not. * denotes *P* < 0.05, Student’s t-test.

In the first set of studies, we tested whether the loss of K_v_4 function alone would affect learning. To do this, we used a transgenic K_v_4 dominant-negative line, *elav-GAL4;;UAS-DNK*_*v*_ *4*, that completely eliminates K_v_4 function [26]. We found that wild-type and the *UAS-DNK*_*v*_ *4* background stock performed similarly, showing a learned preference for the odor associated with fructose during training, with a LI of 0.43 +/- 0.08 and 0.48 +/- 0.05, respectively (Fig. 6A). In contrast, *elav-GAL4;;UAS-DNK*_*v*_ *4* larvae showed no preference for either odor after training (LI = 0.06 +/- 0.15), similar to the known learning mutant *dunce* (*dnc*; LI = 0.08 +/- 0.11) (Fig. 6A). We further tested whether loss of K_v_4 function in MBs alone affected learning/memory function. We used the MB driver *201y-GAL4* and found that in *201y-GAL4;UAS-DNK*_*v*_ *4* larvae, learning was also impaired (LI = 0.0 +/- 0.14; Fig. 6A), suggesting that K_v_4 function in MBs is especially important for learning.

We next tested whether Aβ42-expressing larvae display learning defects. We compared *elav-GAL4;UAS-Aβ42/+* larvae with background control larvae containing either the *elav-GAL4* driver or the *UAS-Aβ42* transgene alone. Larvae were trained, tested and scored as described above. We found that Aβ42-expressing larvae exhibited a severely impaired LI, compared with background controls (LI = -0.03 +/- 0.09 for *elav-GAL4;UAS-Aβ42/+*, LI = 0.23 +/- 0.08 for *UAS-Aβ42/+*, *P* < 0.05; Fig. 6A). We then examined *elav-GAL4;UAS-Aβ42/UAS-K*_*v*_ *4* larvae. We found that over-expression of K_v_4 rescued learning defects in Aβ42-expressing animals, restoring the LI to 0.29 +/- 0.06 (p < 0.05; Fig. 6A). Since all genotypes showed a similar naïve preference for fructose, no naïve preference for AM or OCT, and chemotaxis towards known attractants (S3 Fig.), no defects were likely due to impaired sensory transduction.

To test the specificity of the rescue of Aβ42-induced learning defects by K_v_4, we also expressed *UAS-K*_*v*_ *1*, *UAS-EKO*, or *UAS-CD8-GFP*, in Aβ42-expressing flies. K_v_1 (Shaker) is another A-type K^+^ channel in *Drosophila* that represents a multigene subfamily in mammals. EKO is a genetically modified *Drosophila* K_v_1 channel. EKO channels have been altered so that their activation threshold is more hyperpolarized, near E_K_, and their N-terminal inactivation mechanism has been disabled [40]; expression of EKO has been successfully used as a general suppressor of neuronal activity [40]. We found that the LI of *elav-GAL4;UAS-Aβ42/+* larvae (LI = 0.03 +/- 0.09) was not improved in *elav-GAL4;UAS-Aβ42/K*_*v*_ *1* or *elav-GAL4;UAS-Aβ42/+;UAS-EKO/+* larvae (LI = 0.075 +/- 0.12 and 0.03 +/- 0.10, respectively; Fig. 6A). Expression of *UAS-CD8-GFP*, a non-channel transmembrane protein, also showed no rescue of Aβ42-induced learning defects (Fig. 6A). Thus, the rescue of Aβ42-induced learning defects by K_v_4 appears to be specific, as it could not be replicated with K_v_1 or EKO expression. In addition, these results show that expression of another *UAS* construct (*UAS-Kv1*, *UAS-EKO*, *UAS-CD8-GFP*) did not dilute the effect of *UAS-Aβ42* on learning. Altogether, these studies suggest that Aβ42-induced loss of K_v_4 is likely to be a critical contributor to downstream learning defects.

### Restoration of K_v_4 Levels Rescues Locomotor Defects

Aβ42 expression has previously been shown to result in age-dependent climbing defects [16,18]. Interestingly, we have previously shown that loss of K_v_4 function in *elav;;UAS-DNK*_*v*_ *4* flies results in over-excitation of neurons, loss of reliable repetitive firing, and impaired repetitive behaviors, such as climbing [26]. One possibility is that the Aβ42-induced decrease in K_v_4 contributes to the age-dependent locomotor deficit of Aβ42-expressing flies. So, we examined whether transgenic restoration of K_v_4 was able to rescue the impaired locomotor function seen in Aβ42-expressing flies. Flies were collected over a 2 day period, then aged either 1 or 14 additional days. We used a standard assay that examines the ability of flies to climb against gravity [41,42]; flies’ performance was scored as described (Materials and Methods). At 1–2 days AE, *elav-GAL4;UAS-Aβ42/+* flies scored similarly (1.98 +/- 0.11, n = 10) to background controls (2.25 +/- 0.20 for *elav-GAL4*, 2.57 +/- 0.11 for *UAS-Aβ42/+*, n = 10; Fig. 6B). In contrast, at 14–15 days AE, *elav-GAL4;UAS-Aβ42/+* flies scored significantly lower (0.66 +/- 0.04, n = 10) than background control lines (1.84 +/- 0.15 for *elav-GAL4*, 1.49 +/- 0.09 for *UAS-Aβ42/+*, n = 10; Fig. 6B), as expected. We then examined *elav-GAL4;UAS-Aβ42/UAS-K*_*v*_ *4* flies at 14–15 days. We found that over-expression of K_v_4 increased climbing scores to near wild-type levels (1.63 +/- 0.09, n = 10, *P* < 0.01; Fig. 6B). In contrast, expression of EKO channels or CD8-GFP did not significantly improve climbing scores (0.54 +/- 0.02, n = 10 or 0.71 +/- 0.05, n = 10, respectively; Fig. 6B). These results suggest that the Aβ42-induced loss of K_v_4 channels underlies downstream locomotor defects. Interestingly, over-expression of K_v_1 also rescued the Aβ42-induced climbing score (1.37 +/- 0.60, n = 10; Fig. 6B), suggesting that the A-type channel properties of K_v_1 may be able to substitute for K_v_4 function in locomotor activity.

### Restoration of K_v_4 Slows Aβ42-Induced Neurodegeneration

**Fig 7:**
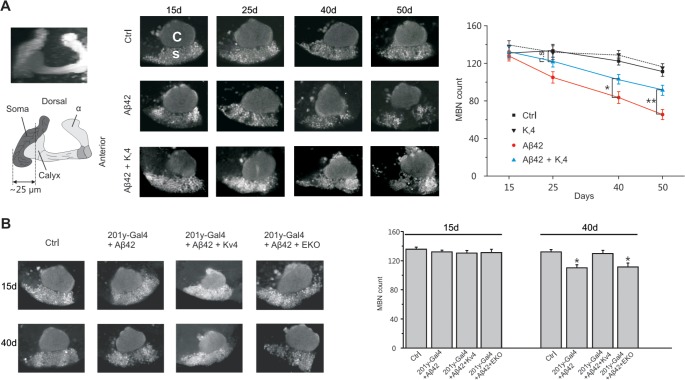
Restoration of excitability in Aβ42-expressing flies ameliorates age-dependent neuronal loss in MBs. (A) Converged confocal z-stack images of a typical mushroom body from the transgenic line, *elav-GAL4;;GFP*.*S65T*.*T10/+* (*Left*, *Top*), and a corresponding diagram with regions containing cell bodies, dendrites (in calyx), and axons shown (*Left*, *bottom*); MB neurons were GFP labeled by the *GFP*.*S65T*.*T10* transgene (regardless of GAL4 driver present, see text) and counted from single images taken 25 μm from the posterior-most edge of MB, as depicted by the dotted line shown. Representative confocal images used for counting neurons are shown from *elav-GAL4;;GFP*.*S65T*.*T10/+* (Ctr), *elav-GAL4;UAS-K*_*v*_ *4/+;GFP*.*S65T*.*T10/+* (K_v_4), *elav-GAL4;UAS-Aβ42/+;GFP*.*S65T*.*T10/+* (Aβ42), and *elav-GAL4;UAS-Aβ42/UAS-K*_*v*_ *4;GFP*.*S65T*.*T10/+* (Aβ42 + K_v_4) flies at indicated ages: 15, 25, 40 and 50 days AE (*Middle*); in confocal images, the calyx (C) is seen above the region containing MB cell somas (s). Quantitative analyses show that restoration of normal excitability by transgenic *UAS-K*_*v*_ *4* expression significantly increases neuronal survival at 40 and 50 days, delaying the onset of neurodegeneration (no significant (n.s,) difference between Ctr and Aβ42 + K_v_4 at 25 d) (*Right*). (n = 6–7 for each group, * *P* < 0.05, ** *P* < 0.01, Student’s t-test). (B) Local expression of *UAS-Aβ42* was induced using the MB driver *201y-GAL4*. Representative confocal images and quantification, as described in (A), are shown from *201y-GAL4;GFP*.*S65T*.*T10/+* (Ctr), *201y-GAL4/UAS-Aβ42/+;GFP*.*S65T*.*T10/+* (201y-Gal4+Aβ42), *201y-GAL4/UAS-Aβ42; GFP*.*S65T*.*T10/UAS-K*_*v*_ *4* (201y-Gal4+Aβ42+K_v_4), and *201y-GAL4/UAS-Aβ42;UAS-EKO/GFP*.*S65T*.*T10* (201y-Gal4+Aβ42+EKO) heads at 15 and 40 days after eclosion. Local expression of Aβ42 results in a significant reduction in MBNs at 40 days (110.30 +/- 4.03), compared to Ctr (132.10 +/- 3.17), which is rescued by K_v_4 expression (129.40 +/- 4.18), but not expression of EKO (111.30 +/- 4.76); n = 8–9 brains for each condition, *P < 0.05, Student’s t-test.

We next investigated whether raising K_v_4 levels in Aβ42 neurons to near wild-type levels, as described above, would attenuate the age-dependent neuronal loss that has been reported to begin at 25 days AE[16]. While secreted Aβ42, and K_v_4, were over-expressed pan-neuronally, we assayed for cell loss specifically in the MBs, using the *GFP*.*S65T*.*T10* transgene. We counted GFP-labeled MB neurons from: 1) a background control line (*elav-GAL4*), 2) the Aβ42-expressing line (*elav-GAL4;UAS-Aβ42/+*), and 3) an Aβ42+K_v_4 line (*elav-GAL4;UAS-Aβ42/UAS-K*_*v*_ *4*). GFP-labeled MB neurons in the intact brain were imaged by confocal microscopy, and cell bodies were counted from images taken at a fixed position (see Fig. 1A) at 15, 25 40, and 50 days AE (aged at 22–23°C). As expected, cell numbers progressively decreased from 25 to 50 days in flies expressing Aβ42 (Fig. 7A). In the Aβ42+K_v_4 line, however, the onset of cell loss was delayed, with no significant degeneration at 25 days (Fig. 7A); at 50 days, cell loss was attenuated by 58% (Fig. 7A). Expression of K_v_4 in a wild-type background (*elav-GAL4;UAS-K*_*v*_ *4/+*) did not increase cell survival (Fig. 7A), suggesting that amelioration of neurodegeneration in Aβ42+K_v_4 brains was indeed due to the restoration of K_v_4.

We also tested whether expression of Aβ42 exclusively in MB neurons would induce local degeneration, and if so, whether this degeneration was also due to hyperactivity generated by the loss of K_v_4. We used the MB GAL4 transgene, *201y-GAL4*, to drive expression of *UAS-Aβ42*, with and without *UAS-K*_*v*_ *4*. In brains from 40 day old flies, we found that the number of MB neurons was reduced by ∼17%, compared to brains from a background control line (Fig. 7B). When *UAS-K*_*v*_ *4* was co-expressed with *UAS-Aβ42*, however, the number of MB cells were similar to controls (Fig. 7B). We also tested if expression of *UAS-EKO* would attenuate Aβ42-induced MB degeneration. The *201y-GAL4* transgene was used to drive MB expression of *UAS-Aβ42*, with and without *UAS-EKO*; flies were aged for 15 and 40 days, then GFP-labeled MB neurons were counted. We found that EKO did not slow Aβ42-induced MB degeneration (Fig. 7B), suggesting that the loss of K_v_4 function, in particular, plays a role in this neuronal degeneration.

**Fig 8:**
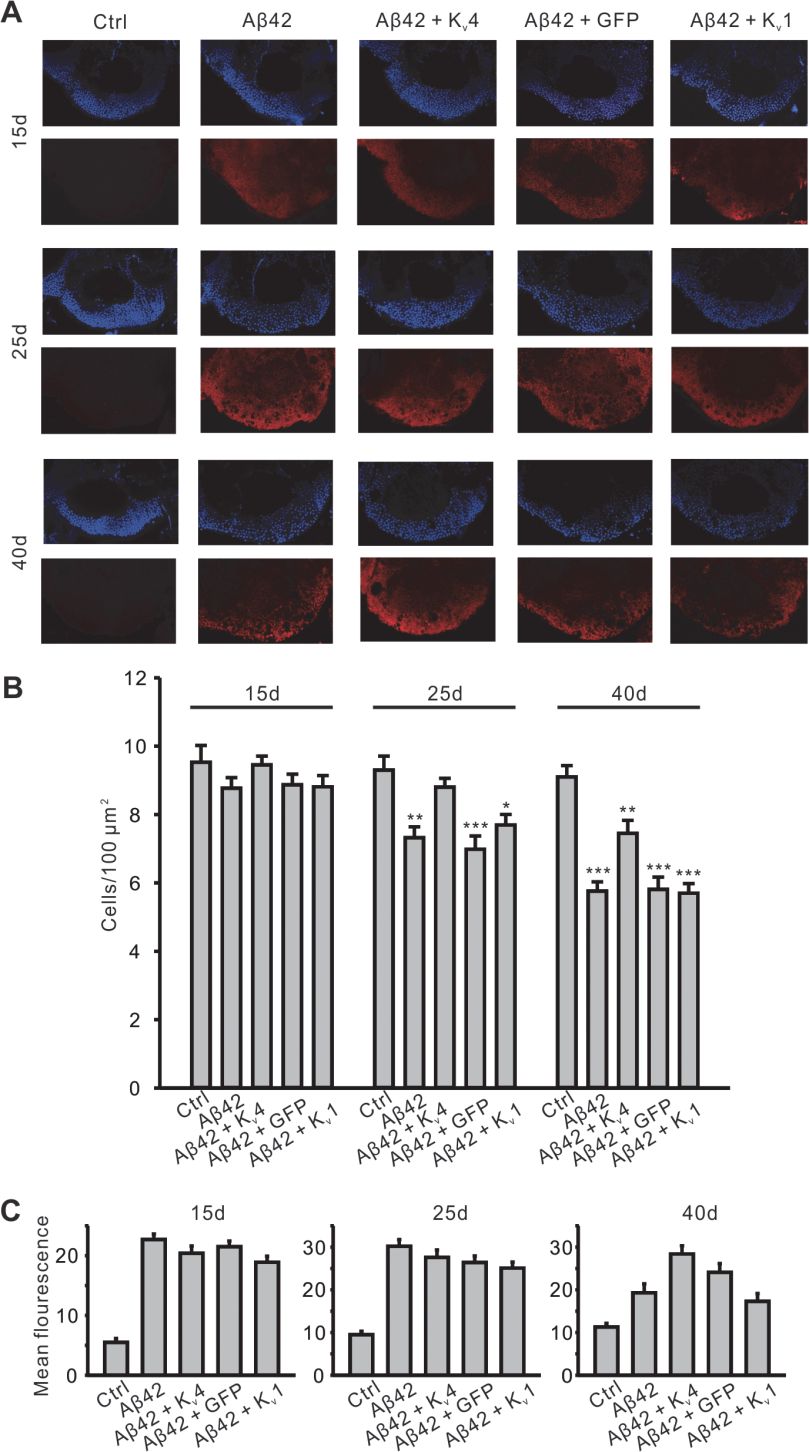
Pan-neuronal expression of K_v_4 slows the onset and severity of Aβ42-induced neurodegeneration. (A) Representative confocal images, from MB position described in Fig. 7, show nuclear (DAPI) staining (upper panels, blue) from five genotypes, including *UAS- Aβ42/+* (Ctr), *elav-GAL4;UAS-Aβ42/+* (Aβ42), *elav-GAL4;UAS-Aβ42/UAS-K*_*v*_ *4* (Aβ42+K_v_4), *elav-GAL4;UAS-Aβ42/UAS-GFP* (Aβ42+GFP) and *elav-GAL4;UAS-Aβ42/UAS-K*_*v*_ *1* (Aβ42+K_v_1). Co-immunolabeling for Aβ42 is shown (lower panels, red) from these genotypes. Brains were dissected and labeled from flies aged 15 days (15d), 25 days (25d) and 40 days (40d) AE. Note that Aβ42 immunostaining is clearly seen in all the genotypes except Ctr. (B) Quantification of cell density (DAPI-positive) from confocal images described in (A). Note that there is significant cell loss in Aβ42, Aβ42+GFP, and Aβ42+K_v_1 flies at 25d, but not in Aβ42+K_v_4. n = 6–8 for each genotype, * P < 0.05, ** P < 0.01, *** P < 0.001, Student’s t-test. (C) Relative anti-Aβ42 signal was quantified in the same aged genotypes described in (A); background fluorescence is reflected in Ctr samples. Note that no significant difference in Aβ42 signal was seen in Aβ42+K_v_4 brains, compared to Aβ42 at 15 and 25 days AE; at 40d AE, Aβ42+K_v_4 brains show an increase in Aβ42 signal; quantification, however, was difficult due to the number of degenerative “holes” seen in confocal sections at this age. n = 6–8 for each genotype. All data are expressed as means ± SEM.

To further examine the specificity of the role of K_v_4 in Aβ42-induced MB degeneration, we investigated the effect of expressing a general *UAS* construct, *UAS-GFP*, or the other A-type K^+^ channel in *Drosophila*, *UAS-K*_*v*_ *1*. In addition, we used another method for quantifying MB neurons and co-labeled for Aβ42 expression. We dissected brains from *elav;UAS-Aβ42/+* flies with and without *UAS-K*_*v*_ *4*, *UAS-GFP*, or *UAS-K*_*v*_ *1* transgenes at 15, 25, and 40 days AE. Brains were co-labeled with DAPI to count cell bodies, and an anti-Aβ42 antibody to compare levels of Aβ42. Brains were imaged by confocal microscopy, as described above, and DAPI-positive cell density was quantified in the MB (Fig. 8A). No cell loss was seen at 15 days AE. Similar to our results quantifying GFP-positive MB cells, we detected a significant loss of DAPI-positive MB cells first at 25 days AE (P <. 01), then continuing at 40 days AE (P < 0.001, Fig. 8B). Over-expression of K_v_4 delayed the onset, and slowed, the ensuing degeneration (Fig. 8B). Since expression of *UAS-GFP* did not attenuate or slow the Aβ42-induced degeneration at 25 or 40 day AE (Fig. 8B), it is unlikely that inclusion of an additional *UAS* target site alone leads to decreased expression of Aβ42 and apparent rescue of neurodegeneration. Indeed, quantification of Aβ42 revealed no loss of Aβ42 expression in Aβ42+K_v_4 MBs (Fig. 8C).

**Fig 9:**
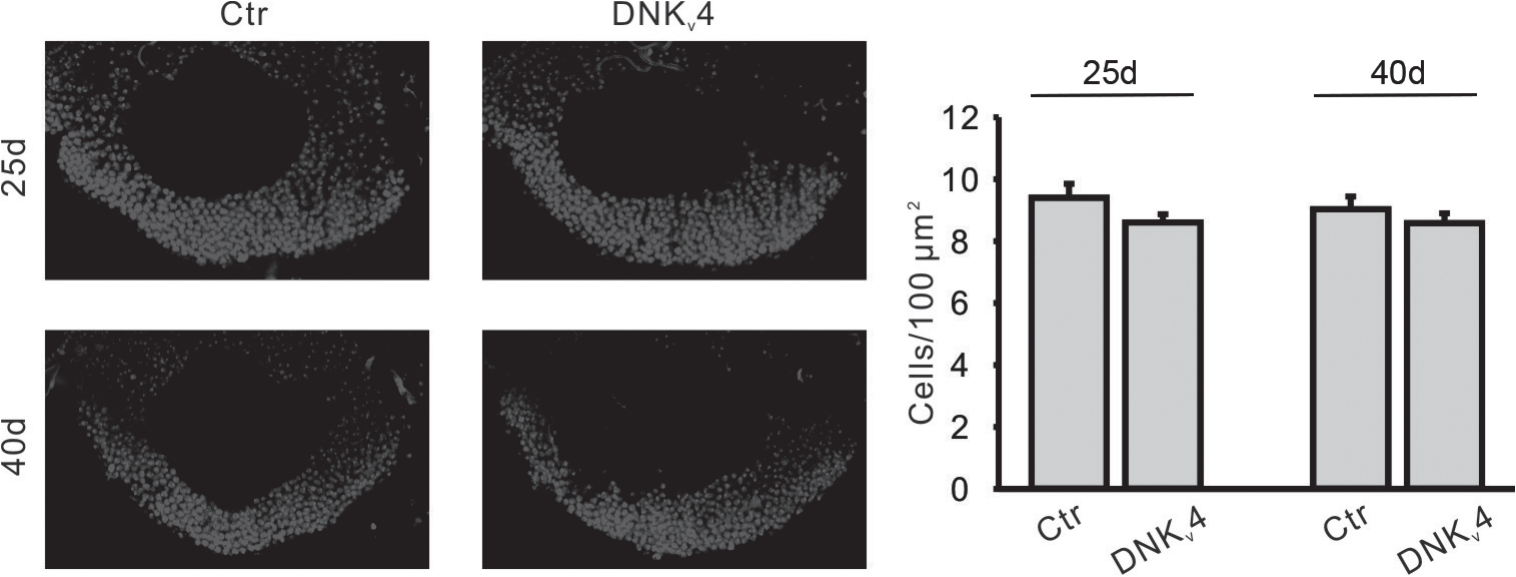
No significant MB cell loss is found in the DNK_v_4 transgenic line. Representative images (*Left*) and quantification (*Right*) of nuclear (DAPI) staining in MBs of *UAS-DNK*_*v*_ *4* (Ctr) and *elav-GAL4;UAS-DNK*_*v*_ *4* (DNK_v_4) at 25 and 40 days AE. MB orientation and position of confocal image as described in Fig. 7. No significant difference in cell density was seen in DNK_v_4 brains compared to controls. n = 6 for each genotype. Data are expressed as means ± SEM.

Interestingly, expression of *UAS-K*_*v*_ *1* resulted in increased cell survival in Aβ42-expressing animals at 25 days AE, but not at 40 days AE (Fig. 8B). These results suggest that A-type K^+^ channel function from K_v_1 can help decrease the severity of initial degeneration, perhaps by ameliorating hyperexcitation. Also interestingly, we found that loss of K_v_4 function alone did not induce neurodegeneration, as observed by DAPI-positive MB cell density in *elav;;UAS-DNK*_*v*_ *4* compared to a background control line at 25 and 40 days AE (Fig. 9). Altogether our results suggest that while the loss of K_v_4 channels, and ensuing hyperexcitability, are key factors that exacerbate neurodegeneration, the Aβ42-induced reduction of K_v_4 channels is not sufficient to trigger degeneration.

### Aβ42-Induced Loss of K_v_4 Contributes to Premature Death

**Fig 10:**
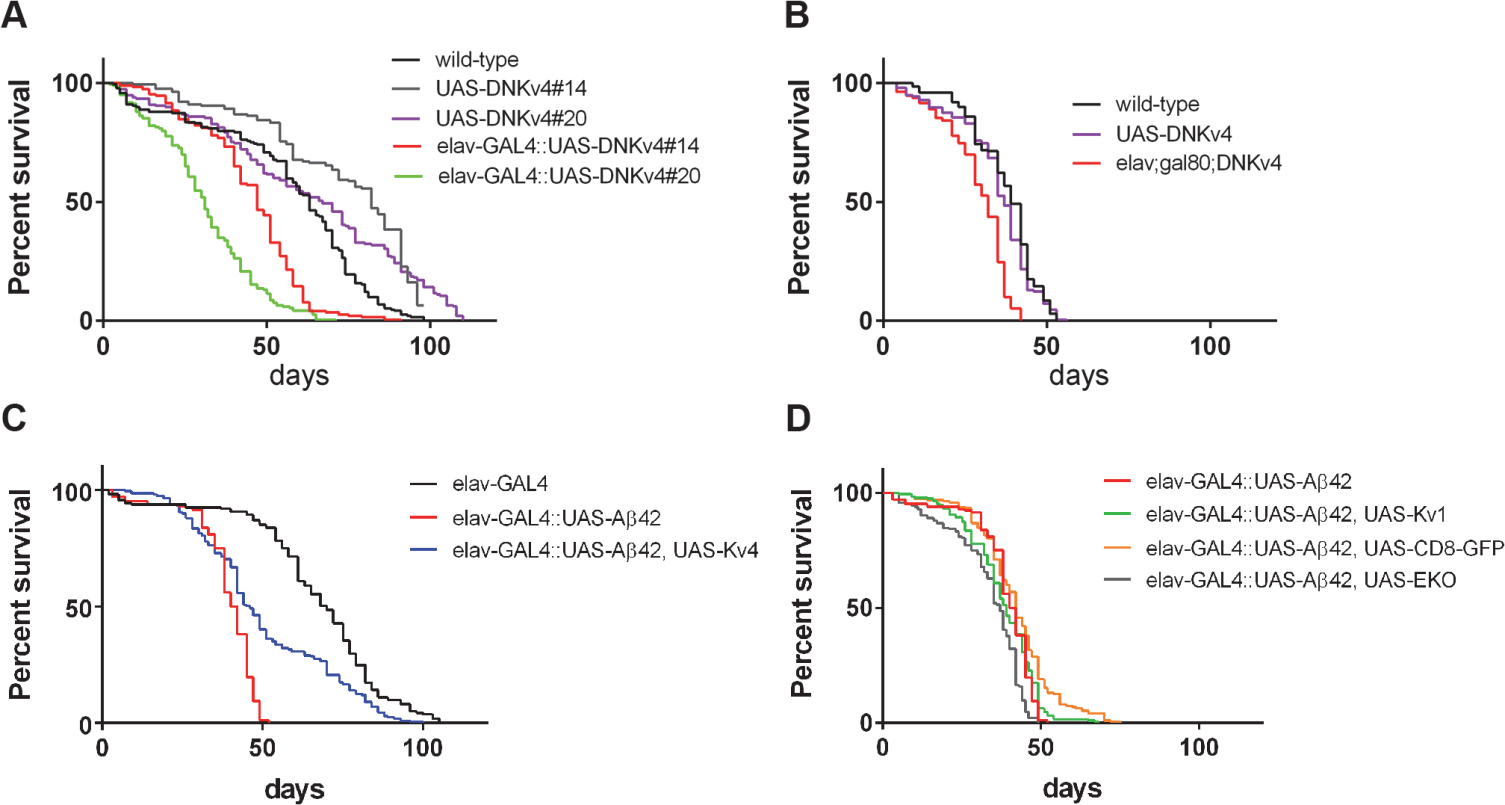
Premature death is partially rescued by expression of K_v_4 in Aβ42-expressing flies. (A-D) Longevity assays, using populations of ∼200 male flies, for indicated genotypes (see Materials and Methods). (A) Survival plots for wild-type, two different transgenic insertions of *UAS-DNK*_*v*_ *4* (#14 and #20), and *elav-GAL4;;UAS-DNK*_*v*_ *4* (elav-GAL4::UAS-DNK_v_4) lines are shown. Neuronal expression of *UAS-DNK*_*v*_ *4* leads to a significantly reduced (*P* < 0.0001 by log-rank analyses) lifespan at 25°C compared to *UAS* background controls. (B) Longevity assays performed for flies raised at 18°C during development, then shifted to 30°C for the entirety of the adult lifespan. Induced expression of DNK_v_4 resulted in a significantly reduced lifespan (*elav-GAL4;tub-GAL80ts;UAS-DNK*_*v*_ *4*; median age of 32 days), compared to wild-type (median age of 39 days) and *UAS-DNK*_*v*_ *4* (median age of 37 days); significance of *P* < 0.0001 by log-rank analyses. (C-D) Survival plots for *elav-GAL4;+* (elav-GAL4), *elav-GAL4;UAS-Aβ42/+* (elav-GAL4::UASAβ42), *elav-GAL4;UAS-Aβ42/UAS-K*_*v*_ *4* (elav-GAL4::UASAβ42,UAS-K_v_4), *elav-GAL4;UAS-Aβ42/UAS-K*_*v*_ *1* (elav-GAL4::UASAβ42,UAS-K_v_1), *elav-GAL4;UAS-Aβ42/+;UAS-CD8-GFP/+* (elav-GAL4::UASAβ42,UAS-CD8GFP), and *elav-GAL4;UAS-Aβ42/+;UAS-EKO/+* (elav-GAL4::UASAβ42,UAS-EKO); all of these assays were performed at 25°C. *elav-GAL4;UAS-Aβ42/+* flies show a severely reduced lifespan (median age of 41 days) at 25°C, compared with the *UAS-Aβ42/+* background control (median age of 70 days; significance of *P* < 0.0001 by log-rank analyses). *elav-GAL4;UAS-Aβ42/UAS-K*_*v*_ *4* flies showed a partial rescue of lifespan (median age of 46 days; P < 0.0001 by log-rank and Gehan-Breslow-Wilcoxon analyses), while *elav-GAL4;UAS-Aβ42/+;UAS-EKO/+*, *elav-GAL4;UAS-Aβ42/K*_*v*_ *1*, *and elav-GAL4;UAS-Aβ42/+;UAS-CD8-GFP/+* flies did not show a significant improvement in lifespan from *elav-GAL4;UAS-Aβ42/+* flies.

Since *elav-GAL4;UAS-Aβ42/+* flies have been shown to exhibit a shortened lifespan [16–18], we examined whether hyperactivity, generated by the loss of K_v_4, might also contribute to this phenotype. We first examined whether loss of K_v_4 function alone would affect lifespan. We compared two different insertions of *UAS-DNK*_*v*_ *4* (#14 and #20), and found that *elav-GAL4;;UAS-DNK*_*v*_ *4* survivorship was significantly decreased, with a median age of 31 and 47 days, compared to 67 and 82 for respective controls (*P* < 0.0001 by log-rank analyses; Fig. 10A). We then restricted expression of *UAS-DNK*_*v*_ *4* to the adult to eliminate effects from loss of K_v_4 function during development. To do this, we used the *UAS-GAL4-GAL80* ^*ts*^ system [43] to induce expression of *UAS-Aβ42* in the nervous system only after adult eclosion. In this system, the GAL80 protein inhibits GAL4 function and therefore Aβ42 expression. We used the *tub-GAL80* ^*ts*^ transgene, which expresses a temperature-sensitive GAL80^ts^ protein under the control of the tubulin promoter; at 18°C, the GAL80^ts^ protein is functional, while a shift to 30°C renders the GAL80^ts^ protein non-functional. Flies containing *elav-GAL4*, *tub-GAL80* ^*ts*^, and *UAS-DNK*_*v*_ *4* transgenes (*elav-GAL4;tub-GAL80* ^*ts*^;*UAS-DNK*_*v*_ *4*) were raised at 18°C to prevent expression of *UAS-DNK*_*v*_ *4*, then shifted to 30°C to induce expression of DNK_v_4 for the lifespan of the adults. Longevity of *elav-GAL4*;*tub-GAL80* ^*ts*^;*UAS-DNK*_*v*_ *4* flies was significantly shorter (median age of 32 days; p < 0.0001) than wild-type (median age of 39 days) and background control (median age of 37 days) lines under similar conditions (Fig. 10B). These results suggest that K_v_4 function in the adult is likely to play a role in longevity.

We next compared the longevity of *elav-GAL4;UAS-Aβ42/+* flies with background control lines. We found that *elav-GAL4;UAS-Aβ42/+* flies have a greatly shortened lifespan (median age of 41 days) compared to the control line (median age of 70 days, p < 0.0001; Fig. 10C). We then tested *elav-GAL4;UAS-Aβ42/UAS-K*_*v*_ *4* flies to see if restoration of normal excitability would attenuate premature death induced by Aβ42. Over-expression of K_v_4 in Aβ42-expressing flies resulted in a small, but significant, increase in lifespan (median age of 46 days, p < 0.05; Fig. 10C). Over-expression of K_v_1, EKO, or CD8-GFP, however, did not significantly increase the lifespan of Aβ42-expressing flies (median ages of 37, 39, and 42 days, respectively; Fig. 10D). These results further support the partial, yet specific, rescue of premature death by K_v_4 expression.

## Discussion

Aβ-induced hyperexcitability is indeed intriguing, with interesting implications especially for seizure-like activity and epilepsy, which are potentially associated with AD [11,15,44,45]. Little, however, has been done previously to determine whether Aβ-induced hyperactivity contributes to downstream behavioral pathologies. Recent studies, however, have suggested that neuronal hyperactivity may precede neurological dysfunction [46] and may be improved by pharmacologically reducing activity [44]. In this study, we show, in cultured neurons and in the intact brain, that K_v_4 channels are specifically down-regulated by Aβ42 expression, while other K^+^ currents (eg. K_v_2 and K_v_3) remain unaltered. The resulting increase in neuronal excitability was present in the adult brain at an age (8 days AE) before the appearance of locomotor (14–15 days AE) and learning defects (14 days AE; ref), and before the onset of neurodegeneration (25 days AE), supporting the hypothesis that hyperactivity precedes and contributes to these downstream pathologies. We then show that increasing K_v_4 channel levels in Aβ-expressing animals restores normal excitability to neurons, and as a result, completely rescues learning and locomotor defects, slows neurodegeneration, and slightly increases lifespan. It is significant to note that the expression of a *UAS-GFP* or *UAS-CD8-GFP* transgene did not rescue any of these pathologies, suggesting that any rescue effects by *UAS-K*_*v*_ *4* were not simply due to the introduction of another *UAS* target for GAL4 that would dilute the expression of Aβ42; indeed, quantification of Aβ42 was not any lower in Aβ42+K_v_4 flies. In future studies, it will be interesting to examine the temporal requirement for reducing excitability with K_v_4 expression; for example, is early hyperexcitability more “toxic” to the system than later stage hyperexcitability?

Although specificity of rescue by K_v_4 varied from one pathology to another, the genetically engineered EKO channel that acts as a general activity inhibitor did not ameliorate any of the cognitive, motor, or survival deficits tested. This suggests that general dampening of excitability was not sufficient to replace K_v_4 loss. K_v_1, the other A-type K^+^ channel present in *Drosophila*, however, was able to rescue Aβ42-induced locomotor dysfunction, but, interestingly, not learning or premature death. These results are consistent with the fact that K_v_1 and K_v_4 share some, but certainly not all, biophysical properties. For example, K_v_4 channels have a much more hyperpolarized voltage-operating range than K_v_1 channels, making them much more likely to play roles at subthreshold potentials [20,21]. Also, while both K_v_4 and K_v_1 channels display fast inactivation, the inactivation rate is voltage-independent for K_v_4 channels and voltage-dependent for K_v_1 channels[20,21]. Finally, the subcellular localization of K_v_4 and K_v_1 channels are thought to be distinct, with K_v_4 channels restricted to dendrites and cell bodies, and K_v_1 channels localized in axons and nerve terminals.

We also examined whether the loss of K_v_4 function alone was sufficient to lead to cognitive and motor pathologies. Previously, we had shown that expression of a dominant-negative K_v_4 subunit, DNK_v_4, results in the elimination of the K_v_4 current[26]. Loss of K_v_4 function led to increased excitability and locomotor deficits[26,47,48]. In the present study, we found that expression of DNK_v_4 also induced learning defects and a shortened lifespan, consistent with a key role for the Aβ42-induced reduction in K_v_4 in these downstream pathologies. In mammalian systems, K_v_4.2 has been shown to play a role in the induction of long-term potentiation (LTP) [49], and hippocampal dependent learning/memory defects [50]. Loss of K_v_4 function alone, however, did not induce any significant neurodegeneration, suggesting that while Aβ42-induced loss of K_v_4 exacerbates degeneration, it is not sufficient to trigger neurodegenerative pathway(s).

Previous reports over the years have shown different effects of Aβ on A-type K^+^ currents *in vitro*, with some identifying decreases in I_A_ [51,52] and others reporting increases in I_A_ [4,53–57]. Differences between these studies are likely to be due to a variety of factors including the species of Aβ tested (eg. Aβ1–40, Aβ1–42, Aβ25–35; some studies finding clear differences with different Aβ species [56], the cell type examined (eg. HEK cells, hippocampal neurons, or cortical neurons), and the time course of the effect (eg. from seconds to days in different studies). For example, the Aβ species applied, the concentration used, and time incubated with cells all affect the assembly state of Aβ, which has also been proposed to have differential downstream effects on K^+^ currents [4,51] and excitability/activity [8,12]. In the future, it will be interesting to see how effects on K_v_4 develop, and possibly change, throughout the assembly of Aβ42 from monomers to oligomers, protofibrils, and mature fibrils *in vivo*.

Much remains to be understood about the mechanism by which K_v_4 channels are lost in response to Aβ42 expression. In this study, pharmacological and genetic approaches suggest a degradation pathway for K_v_4 that depends on both the proteasome and lysosome, similar to the EGF receptor [35,36]. This scenario is likely to be complicated since previous studies have shown that Aβ directly inhibits the proteasome [58], and that clearance of Aβ depends on the proteasome [59–62]. Further study is needed to understand how K_v_4 channels are targeted for turnover by Aβ42, and what other component(s) are involved.

Further study is also needed to unravel specific mechanisms by which K_v_4 channels function in downstream Aβ42 pathologies. For example, how does the loss of K_v_4 exacerbate neurodegeneration? One possibility is that cell death is induced by an “excitotoxic” pathway due to excess Ca^2+^ entry, and ultimately, necrosis. Interestingly, previous studies have shown that Aβ42 induces an increase in various K^+^ currents that are linked to cell death *in vitro* [63–65], consistent with evidence that efflux of K^+^ is required as an early step in apoptosis [66]. While it is not clear how to reconcile these findings with ours, it does seem that proper K^+^ homeostasis is critical for neuronal survival. The role of K_v_4 in lifespan, however, is complex, given that neurodegeneration, learning/memory formation, and locomotor activity all contribute to survival. The partial to full rescue of multiple Aβ42-induced pathologies by K_v_4, however, underscores the importance of the loss of K_v_4 *in vivo* and suggests that K_v_4 is a critical target of Aβ42 in this model, and perhaps in AD.

## Materials and Methods

### Fly Stocks

We used previously generated transgenic lines: *UAS-GFP-K*_*v*_ *4* [67,68], *UAS-Aβ40* and *UAS-Aβ42/CyO* [16–18], *GFP*.*S65T*.*T10* [30,31], *UAS-Pros26* ^*1*^;*UAS-Prosβ* ^*2*^ [32], and *UAS-EKO* [40]. For *UAS-K*_*v*_ *4*, the wild-type *Shal2* isoform was subcloned into the *pENTR1A* vector (Gateway pENTR vectors, Invitrogen), then recombined *in vitro* using lambda integrase into the *pTW* destination vector (*Drosophila* Gateway Vector Collection, available through the *Drosophila* Genomics Resource Center), generating the *pUAST-Shal2* transformation vector. Microinjection with transposase into *w* ^*1118*^ embryos to generate transgenic lines was performed by Rainbow Transgenics (Camarillo, CA), then mapped and balanced by standard procedures. For behavioral studies, fly lines were successively outcrossed at least five times before use.

### Whole-Brain and Embryonic Neuronal Cultures

For whole-brain cultures, flies (2 days after eclosion) were anaesthetized, and the brains were quickly dissected, as reported previously [25]. We incubated the cultures in a humidified chamber at room temperature and refreshed the culture medium (18% fetal bovine serum, 100 U/mL penicillin, 100 μg/mL streptomycin in Schneider’s *Drosophila* culture medium) every 12 hours. 10 brains were sonicated for each immunoblot sample. For embryonic neuronal cultures, single embryos aged 5–6 hours (at room temperature) were dissociated in 20 μL drops of culture medium, as previously described [20,21,25].

### Electrophysiology

Whole-cell recordings were performed in perforated patch-clamp configuration by adding 400–800 μg/ml Amphotericin-B (Sigma-Aldrich) in the pipette, as reported previously [25,26]. For current-clamp recordings, we used external solution (in mM):NaCl, 140; KCl, 2; HEPES, 5; CaCl_2_, 1.5; MgCl_2_, 6, with pH 7.2. For K^+^ current recordings in cultured MB neurons, we used external solution (in mM): Choline-Cl, 140; KCl, 2; MgCl_2_, 6; HEPES, 5, with pH 7.2. For K^+^ current recordings from GFP-labeled MB neurons in the intact brain, we exchanged culture medium to the external solution (in mM): NaCl, 110; KCl, 2; MgCl_2_, 6; glucose, 5; NaHCO_3_, 20; NaH_2_PO_4_. Solution was continuously bubbled with carbogen (5% oxygen and 95% carbon dioxide) throughout recording; pH was set at 7.2. TTX (1 μM) and nifedipine (10 μM) were added to the external solution to block Na^+^ and Ca^2+^ currents. Electrodes were filled with internal solution (in mM): K-gluconate, 120; KCl, 20; HEPEs, 10; EGTA, 1.1; MgCl_2_, 2; CaCl_2_, 0.1, with pH 7.2. We performed all recordings at room temperature. Electrode resistances for all recordings were 5–10 MΩ. Gigaohm seals were obtained for whole-cell recordings.

For analyses shown in Fig. 1D, the correlation coefficient (CC) between the membrane potential and the latency to the first AP was examined. Calculation of this CC was performed as a Pearson product-moment correlation coefficient. The CC is defined by CC = S_XY_ /σ_X_σ_Y_, where σ_X_ and σ_Y_ are the standard deviations (SDs) of the samples, and S_XY_ is the sample covariance of X and Y. Here, X and Y represent the membrane potential and AP latency. The CC for membrane potential and AP latency in wild-type and Aβ42 neurons were quantified, similar to a previous study [69].

### Imaging and Immunoblot Analyses

For fly brain imaging, adult brains were dissected in phosphate buffer (0.1 M, pH 7.2), and fixed for 20 min in 4% paraformaldehyde, as previously described [67]. The isolated brains were washed in phosphate buffer for 5 min, 4 times. We either mounted the brains on the coverslips and captured the images on the same day (for pan-neuronal expression of Aβ42), or blocked in 5% goat serum for 1 hour and immunolabeled with anti-GFP (1:1000) or Anti-Aβ42 (Anti-β-Amyloid (1–42) Rabbit pAb, Calbiochem) in PBST at 4°C overnight (for experiments using the *201y-GAL4* driver). Brains were washed in PBST (5 min, 4 times) and then incubated with secondary antibody for 1 hour at room temperature; brains were washed (5 min, 4 times), then mounted (with DAPI) and imaged. Images were obtained using a Zeiss LSM 510 confocal microscope; images were processed and analyzed using Photoshop CS2 and Volocity v.6 software (Perkin Elmer).

*Neuronal and Aβ42 quantification*. The number of GFP-labeled MB cells and DAPI-labeled cells at MB area were blindly counted by three independent investigators. To quantitate cell density, we chose regions of interest (ROIs) in the area of MB cell bodies of ∼400 μm^2^, and used 3 ROIs from each confocal image. For Aβ42 quantification, grey values of labelled anti-Aβ42 were determined after subtracting the background values, which were taken from non-immunostained brain images. Commonly, the MB area (cell bodies) 1200 μm^2^ in a single section (one from half brain, and two from the whole brain) were picked up for data collection and all data were pooled from around 6–8 brains for each genotype.

For immunoblot analyses, we used primary antibodies at the indicated concentrations, overnight at 21–23°C: anti-K_v_4 (1:100;[68], anti-syntaxin (1:100; 8C3 from Developmental Studies Hybridoma Bank, University of Iowa).

### RT-PCR

Flies were collected, aged at 25°C, and stored at -80°C until ready to extract total RNA. TRIZOL RNA isolation procedure (Invitrogen/Life Science) was used to extract total RNA following homogenization of liquid nitrogen chilled flies in 1.5 ml RNase/DNase–free Eppendorf tubes with 1.5 ml RNase/DNase – free pestle (KONTES-Kimble Chase LLC). The quantity and quality of total RNA was analyzed by NanoDrop, and agarose gel electrophoresis. To eliminate genomic DNA, RNA samples were treated with DNase I. DNase I treated RNA (1 μg) was used as a template to synthesize first-strand cDNA by Superscript II RT (Invitrogen/ Life Science). K_v_4 specific primers were used for standard PCR. Sense primer: AGA ACG GGG ATC AGA ATC TGC A, anti-sense primer: CGG TGG CAA AGA TGA TAA TGG. The quantity of cDNA was measured by Image J following agarose gel electrophoresis. Quantification was averaged over three different total RNA extracts.

### Learning/Memory Assays

Larvae were trained in groups of 5. In each training cycle, larvae were: 1) transferred to an “association plate” in which one of the odors is paired with an agarose-fructose (FRU) substrate for one minute, 2) moved to an agarose-only rest plate one minute, 3) moved to an agarose-only plate with the second odor, and 4) moved to an agarose-only rest plate; 10 such training cycles were performed. After training, larvae were placed on an agarose-only test plate with an AM source on one side, and an OCT source on the other side; after 20 minutes, the number of larvae on each half of the plate were counted. 5–15 groups were assayed for each genotype. Standard preference scores and learning indices were calculated as described in [39]. As an example, the preference score for AM (PREF_AM_), and the learning index (LI) were calculated as shown:

PREF_AM_ = (number of larvae_AM_ – number of larvae_OCT_)/number of larvae_TOTAL_

LI = (PREF_AM+/OCT-_ – PREF_AM-/OCT+_)/2

Given: number of larvae_AM_ or larvae_OCT_ indicates the number of larvae on the AM or OCT, half or the test plate, respectively; PREF_AM+/OCT-_ or PREF_AM-/OCT+_ indicates the preference scores after either AM+/OCT- or AM-/OCT+ training, respectively.

### Locomotion Assays

30–35 males in a 12.4 cm tall tube were allowed to climb upwards for 30 seconds into a second tube inverted on top of the first. The flies that successfully climbed into the second tube were given 30 seconds to climb from the bottom of the tube into a third tube. This process was continued through ten successive tubes and measured by a countercurrent distribution [41]; each fly was given a score of 0.5 for each tube that it climbed out of, similar to [42]. 5–15 assays, with ∼30–35 naïve flies, were performed for each genotype tested.

### Lifespan Analyses

For each genotype, a total population of 200 newly-eclosed male flies were collected and housed in food vials (10 flies/vial) at the indicated temperatures. Food vials were changed every 2–3 days and the number of dead/surviving flies was counted during each change. Log-rank statistical analyses were performed using GraphPad Prism 6 (GraphPad Software, Inc., La Jolla, CA).

## Supporting Information

S1 Fig Summarized results showing the I_A_ and delayed rectifier (DR) currents that are encoded by K_v_4 and K_v_2–3, respectively, from *elav-GAL4* (Ctr) and *elav-GAL4*::*UAS-Aβ42/+* (Aβ42). n = 8. Related to Fig. 2.
(TIF)

S2 Fig Representative blots and quantification of K_v_4 protein levels from *elav-GAL4* (Ctr2) and *elav-GAL4*::*UAS-Aβ42* (Aβ42) heads at indicated ages. Related to Fig. 3.
(TIF)

S3 Fig Olfactory and gustatory assays for genotypes used in learning assays. The following genotypes were tested: wild-type (wt), *UAS-DNK*_*v*_ *4* (UAS-DNK_v_4), *elav-GAL4*::*DNK*_*v*_ *4* (elav-GAL4::DNK_v_4), *elav-GAL4* (elav), *UAS-Aβ42/+* (UAS-Aβ42), *UAS-K*_*v*_ *4* (UAS-K_v_4), *elav-GAL4*::*UAS-Aβ42/+* (elav::Aβ42), *elav-GAL4*::*UAS-Aβ42/+*,*UAS-K*_*v*_ *4* (elav::Aβ42,K_v_4), *dnc* ^*1*^ (dnc), *201y-GAL4* (201y), and *201y-GAL4*::*UAS-DNK*_*v*_ *4* (201y::DNK_v_4). Related to Fig. 6. (A) All genotypes were tested for a natural preference for fructose by placing groups of 5–10 larvae in the center of circular plates with half plain agarose (1%) on one side, and half agarose (1%) + fructose (2M) on the other; after 20 minutes, larvae on each side of the plate were counted and preference scores calculated (n = 3–5 groups for each genotype). All showed a positive preference score for fructose. (B) Larvae were tested for a naïve olfactory preference for AM or OCT, on either plain agarose (AGAR) or agarose+fructose (FRUCTOSE), by placing groups of 5–10 larvae in the center of plates with AM and OCT sources at opposite sides (n = 3–5 groups were used); after 20 minutes, larvae were counted and preference scores calculated for AM/OCT. No genotype showed a strong preference for either odor on plain agarose or fructose. (C) To ensure that larvae could indeed smell, all genotypes were tested as in (B) with a natural attractant (methylacetate or acetone) on one side of the test plate and an empty source at the other. All genotypes showed a positive preference score for the attractants after 20 minutes (n = 3–5 groups of 5–10 larvae).
(TIF)
