# Supplementary figures and images for: Linking Aβ42-Induced Hyperexcitability to Neurodegeneration, Learning and Motor Deficits, and a Shorter Lifespan in an Alzheimer’s Model
Source: PLoS Genet. 2015 Mar 16;11(3):e1005025. doi: 10.1371/journal.pgen.1005025 (PMC4361604; doi:10.1371/journal.pgen.1005025)

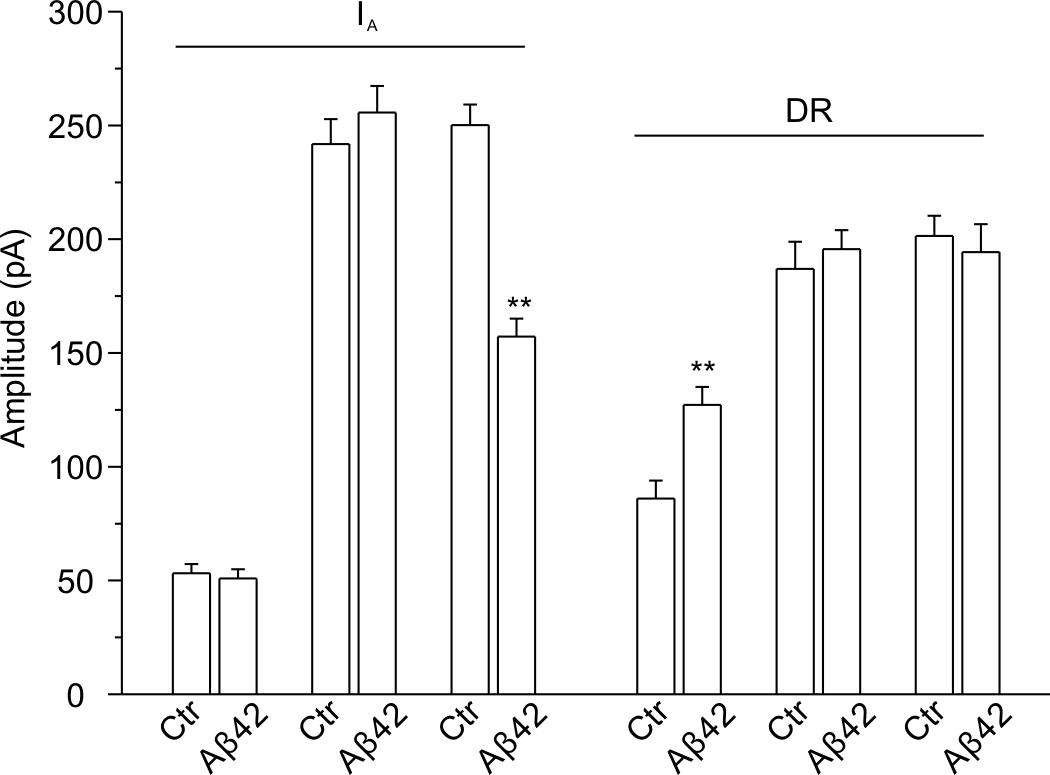

Supplement: S1 Fig — Summarized results showing the IA and delayed rectifier (DR) currents that are encoded by Kv4 and Kv2–3, respectively, from elav-GAL4 (Ctr) and elav-GAL4::UAS-Aβ42/+ (Aβ42). n = 8. Related to Fig. 2. (TIF) [file pgen.1005025.s001.tif]

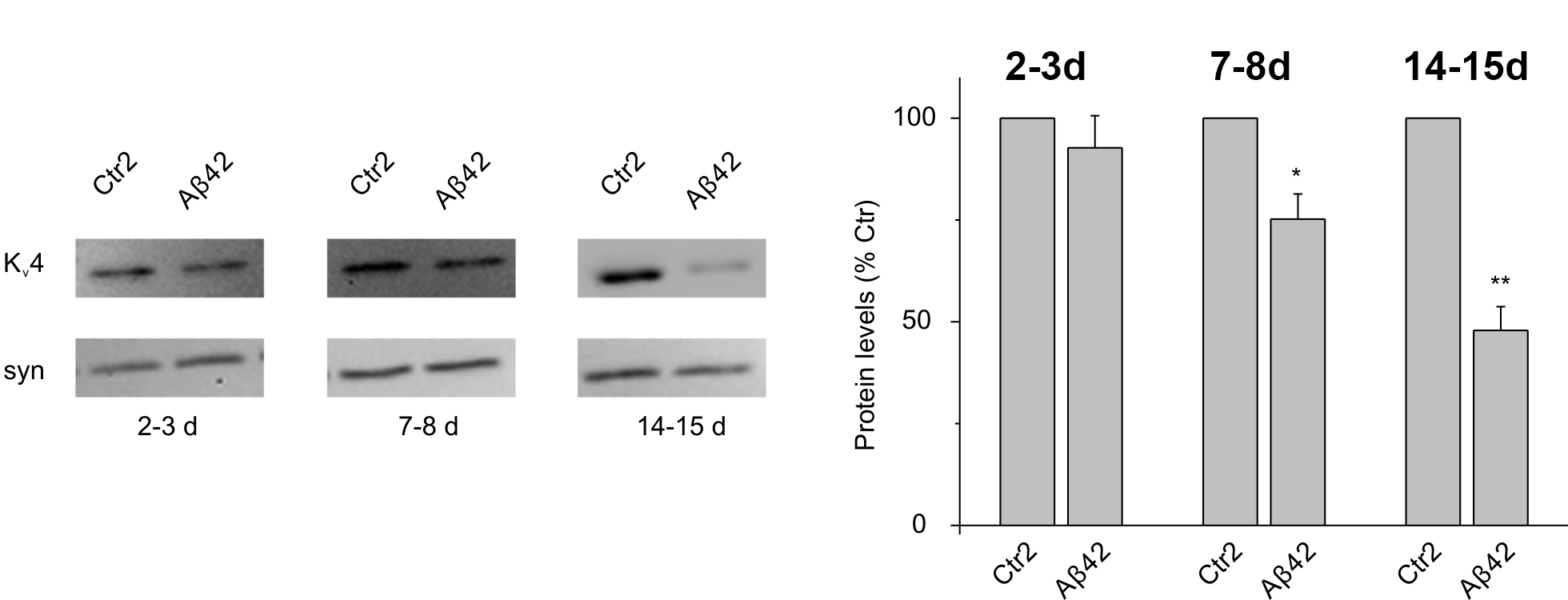

Supplement: S2 Fig — Representative blots and quantification of Kv4 protein levels from elav-GAL4 (Ctr2) and elav-GAL4::UAS-Aβ42 (Aβ42) heads at indicated ages. Related to Fig. 3. (TIF) [file pgen.1005025.s002.tif]

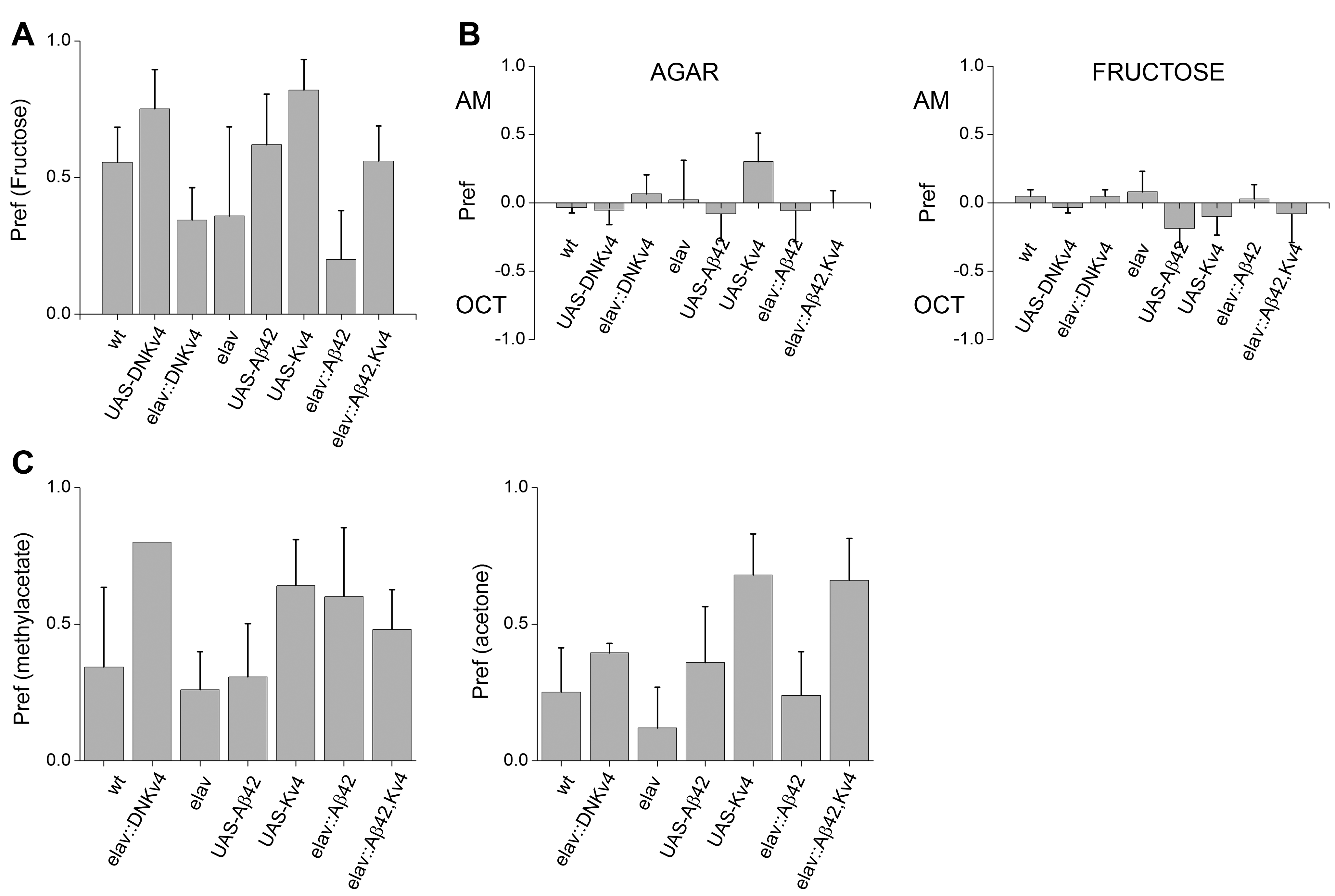

Supplement: S3 Fig — Olfactory and gustatory assays for genotypes used in learning assays. The following genotypes were tested: wild-type (wt), UAS-DNK v 4 (UAS-DNKv4), elav-GAL4::DNK v 4 (elav-GAL4::DNKv4), elav-GAL4 (elav), UAS-Aβ42/+ (UAS-Aβ42), UAS-K v 4 (UAS-Kv4), elav-GAL4::UAS-Aβ42/+ (elav::Aβ42), elav-GAL4::UAS-Aβ42/+,UAS-K v 4 (elav::Aβ42,Kv4), dnc 1 (dnc), 201y-GAL4 (201y), and 201y-GAL4::UAS-DNK v 4 (201y::DNKv4). Related to Fig. 6. (A) All genotypes were tested for a natural preference for fructose by placing groups of 5–10 larvae in the center of circular plates with half plain agarose (1%) on one side, and half agarose (1%) + fructose (2M) on the other; after 20 minutes, larvae on each side of the plate were counted and preference scores calculated (n = 3–5 groups for each genotype). All showed a positive preference score for fructose. (B) Larvae were tested for a naïve olfactory preference for AM or OCT, on either plain agarose (AGAR) or agarose+fructose (FRUCTOSE), by placing groups of 5–10 larvae in the center of plates with AM and OCT sources at opposite sides (n = 3–5 groups were used); after 20 minutes, larvae were counted and preference scores calculated for AM/OCT. No genotype showed a strong preference for either odor on plain agarose or fructose. (C) To ensure that larvae could indeed smell, all genotypes were tested as in (B) with a natural attractant (methylacetate or acetone) on one side of the test plate and an empty source at the other. All genotypes showed a positive preference score for the attractants after 20 minutes (n = 3–5 groups of 5–10 larvae). (TIF) [file pgen.1005025.s003.tif]
